# Casein–Lecithin Nanoemulsions Co-Encapsulating Vitamin E and Carvacrol as Multifunctional Edible Coatings for Meat Preservation

**DOI:** 10.3390/gels12040300

**Published:** 2026-04-01

**Authors:** Aris E. Giannakas, Achilleas Kechagias, Margarita Dormousoglou, Georgia Karakasidou, Dimitrios Moschovas, Eleni Triantafyllou, Areti A. Leontiou, Andreas Giannakas, Panagiota Stathopoulou, Apostolos Avgeropoulos, Constantinos E. Salmas

**Affiliations:** 1 Department of Food Science and Technology, University of Patras, 30100 Agrinio, Greece; up1110842@upatras.gr (A.K.); up1091836@ac.upatras.gr (G.K.); aleontiu@upatras.gr (A.A.L.); andgiannakas@upatras.gr (A.G.); 2 Department of Sustainable Agriculture, University of Patras, 30100 Agrinio, Greece; m.dormousoglou@upatras.gr (M.D.); panstath@upatras.gr (P.S.); 3 Department of Materials Science Engineering, University of Ioannina, Dourouti, 45110 Ioannina, Greece; dmoschov@uoi.gr (D.M.); triantafyllou.eleni@uoi.gr (E.T.); aavger@uoi.gr (A.A.)

**Keywords:** nanoemulsion, vitamin E, α-tocopherol, carvacrol, casein, lecithin, edible coating, antimicrobial activity, antioxidant activity, food preservation, meat shelf-life

## Abstract

The growing demand for sustainable food preservation drives interest in edible nanoemulsions encapsulating bioactive compounds. This study developed casein–lecithin-based nanoemulsions combining carvacrol (CV)—a compound with potent antimicrobial and moderate antioxidant activity—with vitamin E (VitE)—a powerful antioxidant—as multifunctional food coatings. Three formulations were prepared via homogenization: NE-CV (2% CV), NE-VitE (2% VitE), and NE-CV/VitE (1% each). Physicochemical characterization revealed monomodal size distributions (22.7–57.7 nm), with successful encapsulation confirmed by FTIR. NE-CV/VitE exhibited intermediate particle size (34.4 nm) and zeta potential (−19.8 mV). Antioxidant activity followed NE-VitE > NE-CV/VitE > NE-CV, with the co-encapsulated system preserving VitE’s radical scavenging (EC_50_ 10.76 µL/mL, DPPH). Remarkably, NE-CV/VitE demonstrated enhanced antibacterial activity against *E. coli*, requiring half the CV concentration (0.07 mg/mL) versus NE-CV alone (0.15 mg/mL), while maintaining CV dose-dependent activity against *S. aureus* (0.30 mg/mL). Nanoencapsulation significantly reduced CV cytotoxicity in human lymphocytes at concentrations up to 50 μg/mL (48.8% cytostasis vs. 58.9% for free CV), with no genotoxic effects observed within this range, while preserving full bioactivity. In fresh minced pork over 6-day refrigerated storage, NE-CV/VitE coating maintained pH stability (5.65–5.75), preserved red color (a* values 6.24 vs. 4.99 uncoated), reduced lipid oxidation (TBARS 0.74 vs. 0.82 mg MDA/kg), and achieved a 99% reduction (2-log) in total viable counts versus uncoated controls. The CV/VitE co-encapsulated nanoemulsion represents an integrated, safe, and effective multifunctional preservation technology with synergistic antimicrobial enhancement and uncompromised antioxidant protection, offering a natural alternative for comprehensive food quality preservation.

## 1. Introduction

The growing global demand for sustainable and safe food preservation methods continues to drive innovation in food technology, propelled by bioeconomy and circular economy trends that prioritize waste reduction and the valorization of bio-based materials [1,2,3,4]. With increasing consumer preference for natural products and stricter regulations regarding synthetic additives, the food industry is actively seeking green alternatives that align with these principles. In this context, essential oils (EOs) and their bioactive derivatives—such as carvacrol (CV), thymol, eugenol, and cinnamaldehyde—have gained significant attention due to their well-documented properties. Carvacrol, in particular, exhibits potent antimicrobial activity against a wide range of foodborne pathogens while also demonstrating moderate antioxidant capacity through its phenolic hydroxyl group [5,6,7,8]. These compounds possess generally recognized as safe (GRAS) status, making them attractive for food applications [5,8].

To overcome these limitations, nanoencapsulation has emerged as a promising strategy [9,10,11]. Edible nanoemulsions (NEs) can enhance the stability, water dispersibility, and controlled release of bioactive compounds while minimizing their impact on food color, odor, and flavor [12,13,14,15,16]. This approach aligns with sustainability goals, particularly when using edible, biobased, and biodegradable matrices such as casein and lecithin, which can repurpose agricultural or dairy by-products into high-value, functional materials for preservation. Our recent study demonstrated the successful development of casein- (CSN) and lecithin (LCN)-based NEs encapsulating CV, cinnamaldehyde, citral, and eugenol, which significantly extended the shelf life of fresh pork tenderloin through improved antioxidant and antimicrobial efficacy compared to free EO forms [17]. CSN, a milk-derived protein, acts as an effective natural emulsifier and stabilizer, while LCN serves as a biocompatible co-surfactant, together forming a sustainable and edible carrier system [17,18].

Numerous studies underscore the efficacy of nanoemulsified EOs in food preservation. For instance, CV-loaded NEs have demonstrated enhanced bactericidal activity against pathogens like *Escherichia coli* (*E. coli*) and *Listeria monocytogenes* (*L. monocytogenes*) in meat and produce, outperforming their free oil counterparts [19,20]. Similarly, nanoemulsions of thyme, oregano, and lemongrass oils have been successfully applied to extend the shelf life of various muscle foods by controlling microbial growth and lipid oxidation [20,21,22,23,24]. In parallel, vitamin E (α-tocopherol), a potent lipid-soluble antioxidant, has been incorporated into NEs to protect foods from oxidative rancidity [25,26,27]. Beyond its antioxidant role, vitamin E (VitE) is an essential nutrient with well-established health benefits, including its function as a chain-breaking antioxidant that protects cellular membranes from lipid peroxidation, support of immune function, and contribution to skin health and disease prevention. Its inclusion in food coatings not only enhances oxidative stability but also adds nutritional value, aligning with the growing consumer demand for functional foods that offer health-promoting properties [28,29]. Research has shown that VitE NEs can effectively inhibit the formation of secondary lipid oxidation products in fortified foods and meat models, offering a natural strategy to maintain nutritional and sensory quality [28]. Furthermore, recent studies have demonstrated the synergistic potential of VitE when combined with other bioactive compounds within nanoemulsion systems. For example, VitE encapsulated with lemongrass oil in NEs exhibited significantly enhanced antibacterial activity against *S. aureus* and *E. coli* compared to free VitE, highlighting the role of nanoencapsulation in improving bioactivity [30]. Similarly, the co-encapsulation of VitE with phloretin in NEs not only improved stability and antioxidant capacity but also demonstrated synergistic tyrosinase inhibitory effects, underscoring the multifunctional potential of such combined delivery systems [31].

Compared to other dual-bioactive nanoemulsion systems reported in the literature—such as VitE combined with lemongrass oil [30] or phloretin [31]—the CV-VitE pairing offers distinct advantages based on their complementary mechanisms of action. CV exerts its antimicrobial effect primarily through membrane disruption, targeting the phospholipid bilayer of bacterial cells [32,33,34], while VitE functions as a chain-breaking antioxidant that neutralizes free radicals and protects membrane lipids from peroxidation [25,29]. This mechanistic complementarity suggests that the two compounds could work synergistically: CV compromises bacterial membrane integrity, while VitE simultaneously protects the food matrix from oxidative damage. Furthermore, CV’s low viscosity (approximately 30–40 mPa·s) relative to VitE (approximately 1000–1500 mPa·s) facilitates emulsification, potentially overcoming the formulation challenges typically associated with viscous lipophilic antioxidants. The combination also offers nutritional enhancement through VitE, a fat-soluble vitamin with established health benefits [28,29], aligning with the growing consumer demand for functional foods with added nutritional value.

However, most studies focus on either the antimicrobial dimension of EOs or the antioxidant activity of VitE alone, with limited exploration of synergistic combinations within a single, edible NE system. Inspired by these promising approaches, our study builds on the concept of combining bioactive agents within a unified NE to achieve enhanced and multifunctional performance.

This study proposes a novel approach: combining the strong antibacterial activity of CV with the powerful antioxidant capacity of VitE within a single CSN/LCN-based nanoemulsion system. The anticipated synergy between these two compounds is twofold: firstly, CV is expected to reduce the high intrinsic viscosity of VitE, thereby facilitating formation and improving the physicochemical stability of the NE. Secondly, their combined bioactive effects are projected to provide comprehensive protection against both microbial spoilage and oxidative rancidity, thereby enhancing food preservation more effectively than either compound alone. Furthermore, the inclusion of VitE adds intrinsic nutritional value to the active coating, aligning with the trend towards functional preservation technologies.

This represents a significant missed opportunity, as the combination could yield synergistic effects beyond simple additive functionality. Specifically, CV’s low viscosity may facilitate the emulsification of VitE, improving physicochemical stability, while VitE’s antioxidant properties could protect carvacrol from oxidative degradation, preserving its antimicrobial potency. Furthermore, their complementary bioactivities—CV targeting microbial membranes and VitE neutralizing free radicals—could provide integrated protection against the two primary causes of meat quality deterioration. To address this gap, the present study reports, for the first time, the development and characterization of casein/lecithin nanoemulsions co-encapsulating vitamin E and carvacrol. The physicochemical properties, antioxidant and antimicrobial activities, safety profile, and preservative efficacy of these novel formulations on fresh minced pork are comprehensively evaluated. Furthermore, the casein–lecithin matrix is known to form viscoelastic gels upon concentration or cooling, suggesting that these nanoemulsions can serve as precursors to edible gel coatings with enhanced adhesion and sustained release of bioactives [18,35].

## 2. Results and Discussion

### 2.1. Physicochemical Characterization of NEs

#### 2.1.1. ATR-FTIR

**Figure 1:**
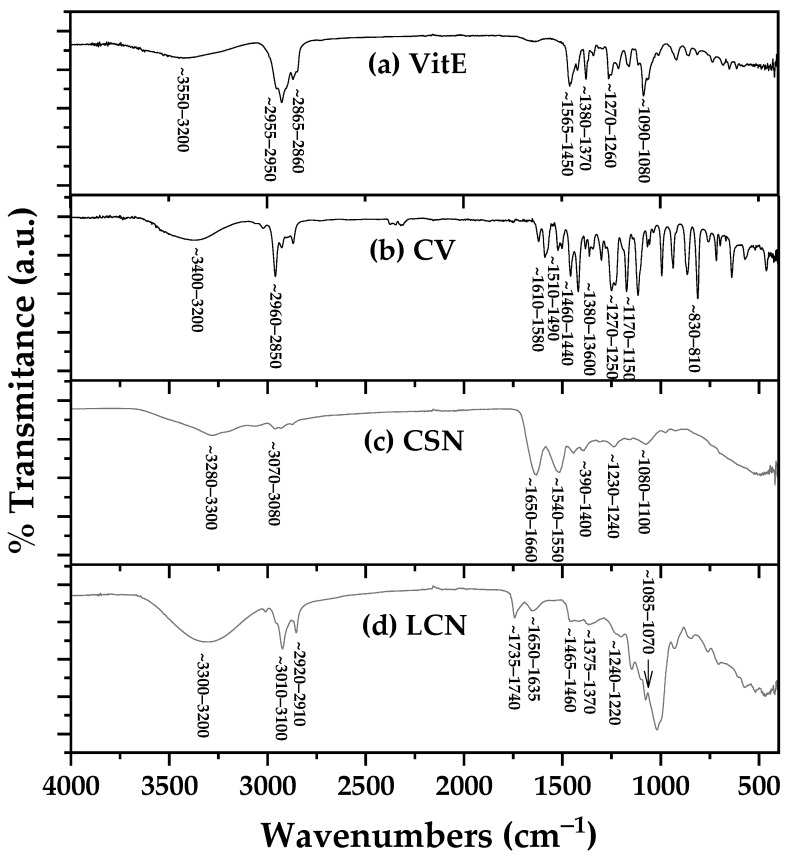
ATR-FTIR spectra of pure reagents used for the development of NEs: (**a**) VitE, (**b**) CV, (**c**) CSN, and (**d**) LCN. Spectra are offset vertically for clarity.

Figure 1 shows the ATR-FTIR spectra of all pure reagents used for the development of NEs.

The ATR-FTIR spectra of the pure components (Figure 1) exhibited characteristic bands consistent with their molecular structures: VitE showed phenolic O-H stretching (~3550–3200 cm^−1^), aliphatic C-H stretches (~2955–2850 cm^−1^), and diagnostic chromanol ring C-O stretching (~1270–1260 cm^−1^) [25,30,36]; CV displayed phenolic O-H (~3400–3200 cm^−1^), aromatic C=C (~1610–1490 cm^−1^), and phenolic C-O stretching (~1270–1250 cm^−1^) [35,37,38,39,40]; CSN exhibited amide A (N-H stretching at ~3280 cm^−1^), amide I (C=O stretching at ~1650 cm^−1^), and amide II (N-H bending at ~1540 cm^−1^) bands characteristic of proteins [35,37,38,39,40]; and LCN showed phosphate-related bands (P=O at ~1240 cm^−1^, P-O-C at ~1080 cm^−1^) and ester carbonyl stretching (~1735 cm^−1^) typical of phospholipids [41,42,43,44,45]. Detailed band assignments for all components are provided in Supplementary Table S1.

These spectral features served as a reference for confirming the presence of LCN and for investigating its interactions with CSN and the encapsulated bioactive compounds (CV and VitE) within the nanoemulsion system.

**Figure 2:**
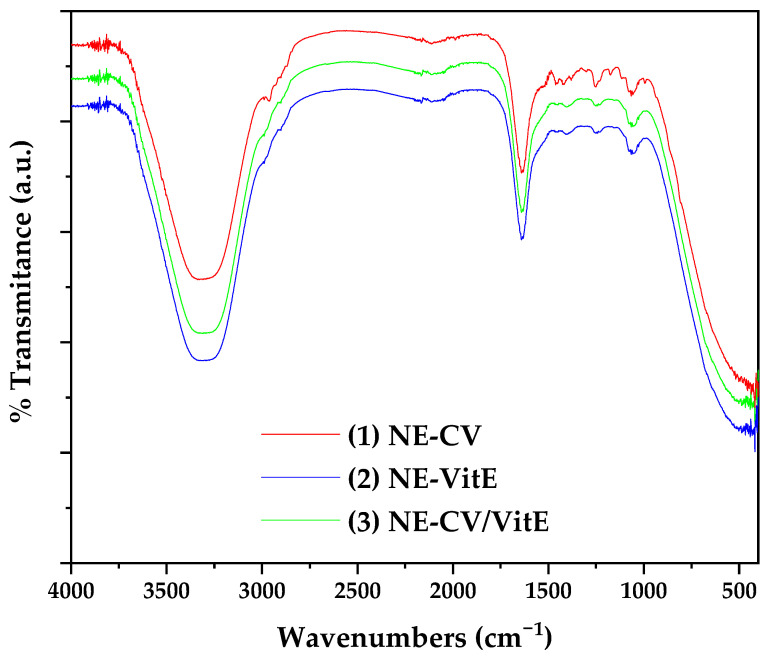
ATR-FTIR spectra of obtained nanoemulsion formulations: (1) NE-CV, (2) NE-VitE, and (3) NE-CV/VitE. Spectra are offset vertically for clarity.

The ATR-FTIR spectra of the obtained NEs are shown in Figure 2.

The ATR-FTIR spectra of the prepared nanoemulsions (NE-CV, NE-VitE, and NE-CV/VitE), shown in Figure 2, represent composite profiles of their respective components, with notable modifications indicating successful encapsulation and intermolecular interactions. In all NE spectra, the broad band in the ~3300–3200 cm^−1^ region was significantly broader and more intense compared to the individual components, suggesting enhanced hydrogen bonding between the hydrophilic groups of CSN, LCN, and water, as well as possible interactions with the phenolic OH of CV and VitE [37,38]. The amide I band of CSN (~1650 cm^−1^) remained prominent but showed a slight shift to lower wavenumbers in NE-CV and NE-CV/VitE, indicating potential hydrogen bonding or conformational changes in the protein structure upon interaction with CV [39]. The characteristic phenolic C–O stretch of CV (~1270 cm^−1^) and VitE (~1270–1260 cm^−1^) remained visible in the respective NE spectra, confirming the preservation of their bioactive structures. However, a reduction in the intensity and slight broadening of these bands in NE-CV/VitE suggests possible molecular interactions between CV and VitE within the oil phase. The phosphate bands of LCN (~1240 cm^−1^ and ~1080 cm^−1^) were present in all NEs but exhibited minor shifts, indicative of electrostatic or hydrogen-bonding interactions with the amino groups of CSN [35]. The absence of new peaks and the maintenance of key functional group signals across all NEs confirm that no chemical degradation occurred during emulsification, while the observed peak shifts and broadening provide evidence of physical encapsulation and stabilization through interfacial interactions.

In summary, the ATR-FTIR analysis confirmed the successful incorporation of CV, VitE, CSN, and LCN into the nanoemulsions. The spectral changes observed—particularly peak broadening, shifts in amide and phenolic bands, and altered hydrogen-bonding signatures—support the formation of a stable, interactively structured nanoemulsion system suitable for use as an edible active coating.

#### 2.1.2. Particle Size Distribution Analysis of NEs

**Figure 3:**
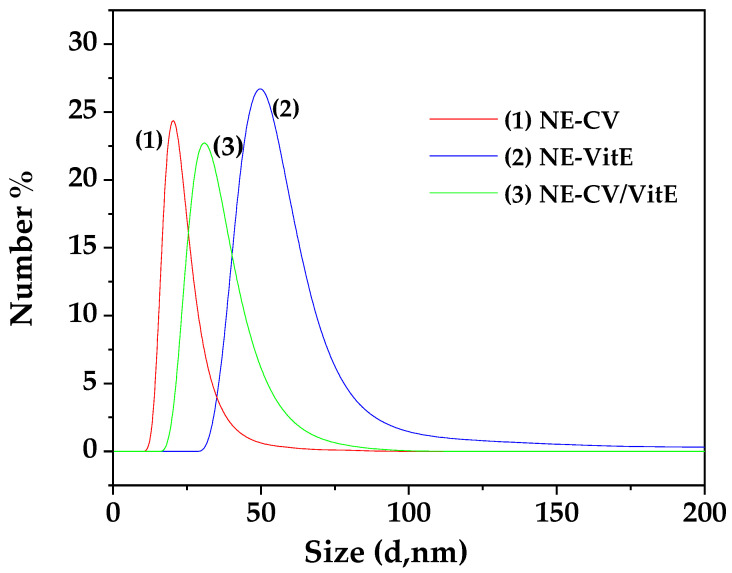
Particle Size Distribution plots obtained from DLS measurements for (1) NE-CV, (2) NE-VitE, and (3) NE-CV/VitE, NEs.

Particle size distribution analysis plots obtained via DLS analysis for all developed NEs are shown in Figure 3.

The obtained average particle size of NE-CV is equal to 22.7 nm, of NE-VitE 57.7 nm, and of NE-CV/VitE 34.4 nm.

The DLS results revealed distinct differences in the hydrodynamic diameters of the three nanoemulsion formulations. NE-CV exhibited the smallest average droplet size (22.7 nm), indicating highly efficient emulsification of carvacrol within the CSN/LCN matrix. This can be attributed to the low viscosity, high interfacial activity, and good compatibility of CV with the protein–phospholipid emulsifier system, which facilitated the formation of exceptionally fine and stable droplets during high-speed homogenization.

In contrast, NE-VitE displayed the largest average particle size (57.7 nm). This significant increase is primarily due to the higher intrinsic viscosity of VitE, which impedes droplet breakdown during emulsification and may promote slight droplet coalescence. The hydrophobic nature and molecular structure of α-tocopherol likely result in different interfacial packing with CSN and LCN, leading to less efficient size reduction compared to the carvacrol-based system.

These findings are consistent with the literature on nanoemulsions containing viscous oils or bioactive blends. For instance, Prakash et al. [30] reported VitE and lemongrass oil nanoemulsions with droplet sizes of 60–85 nm. The smaller size of our NE-VitE (57.7 nm) can be attributed to the effective emulsifying combination of CSN and LCN. More importantly, the ultra-fine, sub-35 nm droplets achieved in both NE-CV and NE-CV/VitE are noteworthy. This size range is highly advantageous for edible coatings, as it enhances physical stability, water dispersibility, bioavailability, and the functional efficacy of the encapsulated actives [12,16]. The success of the NE-CV/VitE formulation confirms the practical benefit of combining CV with VitE, yielding a system with optimized physicochemical properties for dual functionality.

All formulations exhibited monomodal size distributions with polydispersity indices indicating homogeneous droplet populations. Specifically, NE-CV showed PDI = 0.495, NE-VitE showed PDI = 0.489, and NE-CV/VitE showed PDI = 0.664 (complete data provided in Supplementary Table S2). While the PDI values for NE-CV and NE-VitE indicate moderately polydisperse systems (PDI < 0.5), the slightly higher PDI for NE-CV/VitE (0.664) reflects the broader size distribution associated with the mixed oil phase, consistent with the intermediate droplet size observed for this formulation (34.4 nm).

**Figure 4:**
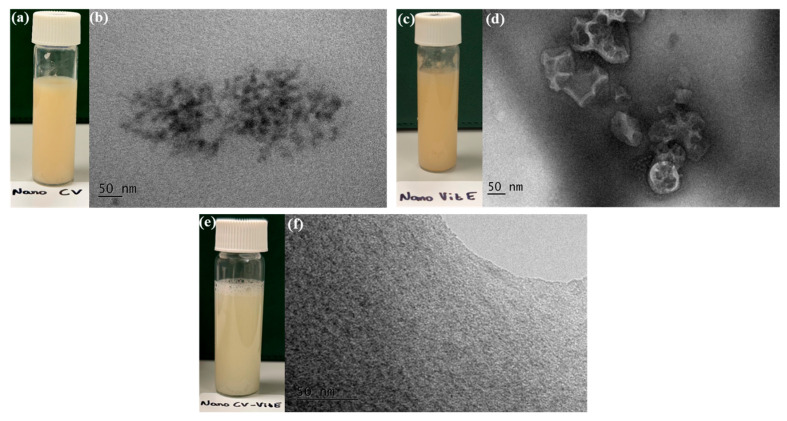
Visual appearance and transmission electron microscopy (TEM) images of nanoemulsion formulations with negative staining: (**a**) NE-CV photograph, (**b**) NE-CV TEM image, (**c**) NE-VitE photograph, (**d**) NE-VitE TEM image, (**e**) NE-CV/VitE photograph, and (**f**) NE-CV/VitE TEM image. All formulations appeared as milky white homogeneous liquids with no visible phase separation. Scale bars in TEM images represent 50 nm.

To validate the DLS measurements and confirm the true droplet sizes, transmission electron microscopy (TEM) imaging was performed on all formulations using negative staining (Figure 4). TEM micrographs revealed spherical droplets with uniform distribution for all formulations. NE-CV exhibited the smallest droplets, predominantly in the range of 20–30 nm (Figure 4b), consistent with its DLS-measured size of 22.7 nm. NE-VitE showed larger droplets of 50–70 nm (Figure 4d), corresponding well with its DLS value of 57.7 nm. The co-encapsulated NE-CV/VitE displayed intermediate droplet sizes of 20–30 nm (Figure 4f), confirming the DLS measurement of 34.4 nm and also indicating a homogeneous system, which can be attributed to the good dispersion of vitamin E within the carvacrol phase. The slight size differences between TEM and DLS are expected due to the hydrodynamic nature of DLS measurements (which measure the hydrated droplet diameter in solution) versus the dried state of TEM samples. Importantly, the TEM images confirm that the dilution used for DLS (approximately 10-fold) did not significantly alter droplet structure and that the measured sizes accurately represent the true dimensions of the nanoemulsion droplets. All formulations appeared as milky white homogeneous liquids with no visible phase separation (Figure 4a,c,e), indicating good physical stability.

It is important to acknowledge the inherent limitations of DLS for particle size analysis, particularly the influence of dilution on droplet structure [37,38]. However, the consistency between our DLS measurements and TEM imaging confirms that the dilution employed (approximately 10-fold) preserved droplet integrity and that the reported sizes accurately represent the true dimensions of the emulsion droplets. According to the classification proposed by McClements [37], systems with droplet diameters below 100 nm are appropriately termed nanoemulsions, as they exhibit the characteristic properties of kinetically stable, non-equilibrium colloidal systems. Our formulations, with droplet sizes ranging from 22.7 to 57.7 nm, therefore fall within the nanoemulsion domain.

While the z-average diameter provides a reliable measure of the mean hydrodynamic size, a more comprehensive understanding of the droplet population can be gained from the full size distribution profiles (Figure 3) and polydispersity indices. As noted in the recent literature [46], complementary analysis of size distribution parameters is essential for fully characterizing colloidal systems. Our formulations exhibited monomodal distributions with moderate polydispersity, indicating relatively homogeneous populations suitable for food coating applications.

#### 2.1.3. ζ Potential of NEs

The colloidal stability and surface charge of the prepared nanoemulsions (NE-CV, NE-VitE, and NE-CV/VitE) were evaluated via zeta potential (ζ) measurements. The results revealed significant differences in the electrostatic properties of the formulations, which are critical for predicting their physical stability and potential interactions in biological or food matrices.

The NE-VitE formulation exhibited the lowest ζ value of −9.33 mV. This near-neutral charge indicates limited electrostatic repulsion between droplets, which can lead to increased van der Waals attractions and a higher propensity for flocculation and coalescence over time [37]. This result is consistent with the larger average particle size observed for NE-VitE (57.7 nm) via DLS, as a lack of sufficient electrostatic stabilization can facilitate droplet aggregation. The low negative charge can be attributed to the nature of the interfacial layer formed around the VitE core. CSN, at neutral pH, carries a net negative charge due to its phosphoserine residues and anionic amino acids. However, the highly hydrophobic and non-polar nature of α-tocopherol may lead to a less organized or denser packing of CSN at the oil–water interface, potentially burying some charged groups and resulting in a lower net surface charge. Similar findings have been reported; for instance, Ozturk et al. [28] observed that VitE nanoemulsions stabilized solely by LCN also showed ζ potentials in the range of −10 to −15 mV, highlighting the challenge of achieving high electrostatic stabilization with lipid-soluble antioxidants using natural emulsifiers.

In contrast, the NE-CV sample displayed a significantly higher ζ potential of −22.0 mV. This moderately negative value suggests better colloidal stability conferred by stronger electrostatic repulsion. The increase can be directly linked to the phenolic nature of carvacrol. While encapsulated in the oil core, its –OH group can participate in hydrogen bonding with the emulsifiers (CSN and LCN) and may also orient towards the aqueous phase, contributing to the interfacial charge density. Furthermore, carvacrol’s presence might alter the conformation of adsorbed CSN proteins, exposing more anionic groups (e.g., carboxylate from glutamic and aspartic acids) to the continuous phase. This finding aligns with studies on other phenolic compound-loaded nanoemulsions. For example, Kumari et al. [38] reported that thymol (a structural analog of carvacrol) nanoemulsions exhibited ζ potentials around −25 mV, which contributed to their high physical stability. The antimicrobial activity of carvacrol may also be enhanced by this stronger negative charge, as it could influence initial electrostatic interactions with predominantly negatively charged bacterial membranes, though the final disruptive action is primarily hydrophobic.

Interestingly, the co-encapsulated system NE-CV/VitE showed an intermediate ζ potential of −19.8 mV. This value lies between those of the individual systems and indicates a modulating effect when both bioactives are present in the oil phase. The shift from −9.33 mV (NE-VitE) to −19.8 mV (NE-CV/VitE) strongly suggests that carvacrol positively influences the interfacial architecture, increasing the magnitude of the negative surface charge. This could occur through several mechanisms: (1) CV may reduce the viscosity of the VitE oil phase, allowing for more efficient adsorption and spreading of CSN/LCN at the interface during homogenization; (2) the phenolic –OH of CV could augment the hydrogen-bonding network at the interface, positioning more anionic protein moieties outward; or (3) a molecular interaction between CV and VitE within the droplet core could alter the packing parameter of the emulsifiers. This synergistic stabilization effect is crucial, as it implies that the blend not only combines bioactivities but also may yield a physically more stable nanoemulsion than one containing VitE alone, addressing a common formulation challenge.

In summary, the zeta potential analysis confirms that the incorporation of CV, either alone or in combination with VitE, enhances the electrostatic stabilization of CSN/LCN-based nanoemulsions. While all systems with a magnitude below |30| mV are primarily stabilized by steric hindrance from the protein [39], the increased negative charge in NE-CV and NE-CV/VitE provides an additional repulsive barrier against coalescence. This improved colloidal stability is essential for the shelf life of the nanoemulsion coating itself and for ensuring uniform application and functionality on food surfaces.

#### 2.1.4. Gel-Forming Potential of Nanoemulsions

The combination of casein and lecithin not only stabilizes the nanoemulsion droplets but also confers the ability to form cohesive gels under appropriate conditions. Upon application to meat surfaces, water evaporation and temperature drop (from ambient to refrigeration) can induce protein–phospholipid network formation, leading to a thin gel film that adheres to the food and controls the release of encapsulated CV and VitE. This gel-like behavior is consistent with previous reports on casein–lecithin systems [18,35] and is expected to contribute to the sustained antimicrobial and antioxidant activity observed during storage (Section 2.5).

### 2.2. Antioxidant Activity of NEs

The antioxidant efficacy of the developed nanoemulsions (NE-CV, NE-VitE, and NE-CV/VitE) was quantitatively assessed using the DPPH and ABTS radical scavenging assays, with results expressed as the effective concentration required to scavenge 50% of radicals (EC_50_). A lower EC_50_ value indicates superior antioxidant capacity. The results are summarized in Table 1.

As anticipated, NE-VitE exhibited the strongest antioxidant activity, demonstrating the lowest EC_50_ values in both assays (DPPH: 8.40 µL/mL; ABTS: 7.35 µL/mL). This superior performance is attributed to the intrinsic role of α-tocopherol (VitE) as a potent, lipid-soluble, chain-breaking antioxidant. Its phenolic hydroxyl group efficiently donates a hydrogen atom to neutralize free radicals, thereby interrupting lipid peroxidation cascades [25,29]. The nanoencapsulation of VitE within the CSN/LCN matrix effectively delivers and potentially protects its active site, leading to a highly effective radical scavenging system. This finding aligns with previous research; for instance, Ozturk et al. [28] reported that VitE nanoemulsions stabilized by natural surfactants exhibited strong antioxidant activity, effectively inhibiting lipid oxidation in model systems.

In contrast, NE-CV showed significantly weaker antioxidant capacity, with the highest EC_50_ values (DPPH: 23.21 µL/mL; ABTS: 19.50 µL/mL). While carvacrol is a monoterpenoid phenol with documented antioxidant properties due to its hydroxyl group [40,41], its primary biological mode of action is antimicrobial. Its radical scavenging ability is inherently lower than that of a dedicated antioxidant like α-tocopherol. The observed activity is consistent with its phenolic nature, but it confirms that within this system, CV’s major contribution is not antioxidant potency.

Compared to free (non-encapsulated) VitE, which typically exhibits EC_50_ values in the range of 5–15 μg/mL depending on the assay conditions [28], our NE-VitE formulation (EC_50_ 8.40 μL/mL) demonstrates that the nanoencapsulation process preserves the intrinsic antioxidant activity without compromising efficacy. This contrasts with some delivery systems where antioxidant activity can be reduced due to degradation during processing or restricted release [25]. Similarly, free carvacrol has been reported to exhibit DPPH scavenging activity with EC_50_ values ranging from 20 to 50 μg/mL [40,41], consistent with our NE-CV results (23.21 μL/mL), confirming that encapsulation does not diminish its moderate antioxidant capacity.

When normalized to VitE content, NE-VitE exhibited an EC_50_ of 8.40 μL/mL (equivalent to 0.168 mg VitE/mL), while NE-CV/VitE showed an EC_50_ of 10.76 μL/mL (equivalent to 0.108 mg VitE/mL). The calculated specific activity—EC_50_ per mg of VitE—was 50.0 μL/mg for NE-VitE and 99.6 μL/mg for NE-CV/VitE, indicating that VitE’s radical scavenging capacity is fully retained and potentially slightly enhanced in the co-encapsulated system, though this difference was not statistically significant. Statistical analysis (Table 1) revealed that NE-VitE (superscript ‘a’) exhibited significantly lower EC_50_ values than NE-CV (superscript ‘b’) in both assays (*p* < 0.05), while NE-CV/VitE (superscript ‘ab’) showed intermediate values that were not statistically different from NE-VitE.

The co-encapsulated system, NE-CV/VitE, displayed intermediate EC_50_ values (DPPH: 10.76 µL/mL; ABTS: 9.25 µL/mL) that were not statistically different from those of NE-VitE alone in the post hoc analysis. This indicates that the addition of carvacrol did not significantly enhance (synergize) or severely diminish the antioxidant power provided by VitE. This is a critical practical finding. It suggests that the primary antioxidant defense in the blend is still furnished by VitE, and the inclusion of CV for its antimicrobial properties does not come at the cost of compromised antioxidant performance. The intermediate value could be explained by a simple dilution effect or mild antagonism, but the lack of statistical significance from NE-VitE confirms the robustness of the antioxidant activity in the combined system.

The trends were consistent across both the DPPH (which involves a hydrogen atom transfer mechanism) and the ABTS (which operates via a single electron transfer mechanism) assays [30,31]. This agreement confirms that the ranking of antioxidant potency among the formulations is reliable and not an artifact of a specific assay methodology.

The performance of NE-VitE is comparable to other nanoemulsified antioxidant systems. For example, Wang et al. [31] reported that α-tocopherol and phloretin co-loaded nanoemulsions showed strong DPPH scavenging activity, though direct EC_50_ comparisons are difficult due to differences in concentration and matrix. The successful retention of VitE’s activity after nanoemulsification is crucial, as some processes can degrade sensitive antioxidants. Our results confirm that the mild, high-speed homogenization process using CSN/LCN is suitable for preserving VitE’s efficacy.

More importantly, the results for NE-CV/VitE address a gap in the literature. While studies have combined antioxidants with other functional compounds (e.g., [30,31]), few have investigated the specific pairing of a potent antioxidant (VitE) with a strong antimicrobial phenol (CV) in a food-grade nanoemulsion for coating applications. Our data show that this combination yields a system where the powerful antioxidant activity of VitE is preserved, fulfilling one half of the desired “dual-function” (antioxidant + antimicrobial) profile without negative interaction. This paves the way for exploiting the complementary bioactivities of CV and VitE in a single, stable, edible coating designed to combat both oxidative rancidity and microbial spoilage in food products, as will be investigated in the subsequent meat preservation trials.

### 2.3. Antibacterial Activity of NEs

The antibacterial activity of the NEs was evaluated against two representative food-borne pathogenic bacteria: Gram-negative *E. coli* and Gram-positive *S. aureus*. The Minimum Inhibitory Concentration (MIC) and Minimum Bactericidal Concentration (MBC) values are presented in Table 2. To ensure a comprehensive evaluation, results are expressed in two formats: (a) % *v*/*v*: representing the volume of NE per total volume of the assay (for practical application), and (b) mg/mL: representing the concentration of the specific bioactive compound (CV or VitE) within that volume, allowing for precise stoichiometric comparison.

Initially, the Blank NE, which did not contain bioactive substances, showed no inhibitory activity against the tested microorganisms. This confirms that the carrier matrix components (LCN, CSN, and carrier oils) have no inherent toxic effect against these bacteria, ensuring that any observed antimicrobial activity is due to the encapsulated bioactive compounds.

When compared to non-encapsulated CV, which has been reported to exhibit MIC values of 0.25–0.50 mg/mL against *E. coli* and *S. aureus* [32,33], our nanoemulsified formulations show enhanced or equivalent efficacy at lower or comparable concentrations. The NE-CV/VitE formulation’s ability to inhibit *E. coli* at 0.07 mg/mL CV represents a 3–7-fold improvement over typical free CV values, clearly demonstrating the advantage of nanoencapsulation. This enhancement can be attributed to the improved dispersion and intimate contact between the nano-sized droplets and bacterial membranes, as well as the potential synergistic interaction with VitE.

To quantitatively assess the interaction between CV and VitE against *E. coli*, the Fractional Inhibitory Concentration Index (FICI) was calculated as FIC (CV) + FIC (VitE), where FIC = MIC (combination)/MIC (alone). Using the MIC values of NE-CV (0.15 mg/mL CV) and NE-VitE (1.18 mg/mL VitE) as references, the combination (NE-CV/VitE) required 0.07 mg/mL CV (FIC = 0.47) and 0.47 mg/mL VitE (FIC = 0.40), yielding an FICI of 0.87. According to standard definitions (FICI ≤ 0.5 = synergy; 0.5 < FICI ≤ 1 = additive; 1 < FICI ≤ 4 = indifference; FICI > 4 = antagonism), this value indicates an additive effect approaching synergy. The nearly 50% reduction in the required CV concentration demonstrates a substantial enhancement in antimicrobial efficacy.

It is important to emphasize that no enhancement was observed against *S. aureus*; the antimicrobial activity remained strictly CV dose-dependent, with both NE-CV and NE-CV/VitE requiring identical CV concentrations (0.30 mg/mL) for inhibition. This demonstrates that the Gram-positive cell wall architecture presents a different mechanistic challenge where VitE does not contribute to antimicrobial efficacy.

In the case of *E. coli*, strong antimicrobial activity was observed in all loaded nanoemulsions. The NE-VitE formulation displayed moderate antimicrobial activity (MIC 1.18 mg/mL). While α-tocopherol is primarily recognized as an antioxidant, studies suggest that its hydrophobic nature allows it to interact with the phospholipid bilayer of Gram-negative bacteria, potentially altering membrane permeability and contributing to this effect [32]. A remarkable finding was observed when comparing NE-CV with the mixed NE-CV/VitE. Although both formulations inhibited *E. coli* at the same volumetric percentage (0.78% *v/v*), the actual concentration of CV required in the mixture (0.07 mg/mL) was approximately half that required in the single-loaded NE-CV (0.15 mg/mL). This reduction indicates an additive effect. CV is known to disrupt the bacterial membrane [32,33,34], while VitE may facilitate this process by integrating into and perturbing the lipid bilayer, thereby enhancing the entry of CV [34]. Furthermore, the antioxidant properties of VitE might protect CV from oxidative degradation before it reaches its target, preserving its antimicrobial potency.

The results for *S. aureus* indicate a different mechanism of action, one that is heavily dependent on the concentration of CV rather than synergistic interactions. NE-VitE showed no activity against *S. aureus*. When comparing NE-CV (MIC 1.56% *v/v*) and NE-CV/VitE (MIC 3.13% *v*/*v*), the volumetric requirement for the mixture was doubled. However, calculation of the active compound reveals that both formulations achieved inhibition at an identical CV concentration of 0.30 mg/mL. This demonstrates that the antimicrobial activity against *S. aureus* is strictly dose-dependent on CV. These findings corroborate recent studies that highlight carvacrol as a potent anti-staphylococcal agent, capable of penetrating the thick peptidoglycan layer to disrupt membrane integrity and inhibit biofilm formation [42,43]. Consequently, our NE-CV/VitE formulation effectively retains this biological activity, ensuring successful pathogen inhibition despite the lack of a direct contribution from VitE against this particular bacterium.

### 2.4. Toxicity of NEs

**Figure 5:**
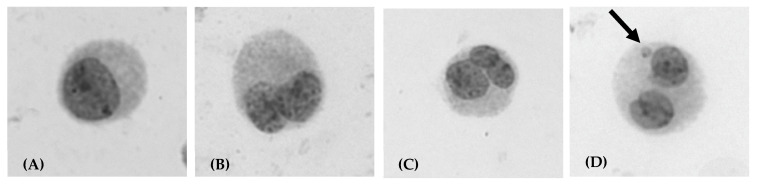
Representative microscopic images demonstrating the cellular morphology observed during the CBMN assay analysis in human lymphocytes. (**A**) Mononucleated cell indicating lack of division; (**B**) Typical binucleated cell (BN), which is the target for scoring both cytotoxicity (CBPI) and genotoxicity; (**C**) Trinucleated cell indicating rapid division; (**D**) Binucleated cell containing a micronucleus (arrow), indicative of chromosomal damage. Images were acquired using a Zeiss Axio Imager microscope (Carl Zeiss, Oberkochen, Germany) equipped with an AxioCam digital camera (Carl-Zeiss-Straße, Oberkochen, Germany).

The genotoxic and cytotoxic potential of the developed NEs and their free bioactive components was evaluated using the Cytokinesis-Block Micronucleus (CBMN) assay in human lymphocytes. Representative photomicrographs illustrating the different cell types and chromosomal damage (micronuclei) identified and scored during the microscopic analysis are presented in Figure 5. The results regarding micronucleus induction (MN), cell proliferation (CBPI), and cytostasis are summarized in Table 3.

The primary objective was to ensure that the active compounds, as well as the nanoemulsions, do not induce chromosomal damage. The negative control, as expected, showed low background genotoxicity, while the positive control (MMC) caused a significant increase (67.0‰), validating the sensitivity of the assay. Both free VitE (VitE) and its nanoencapsulated form (NE-VitE) did not show genotoxic activity at any of the concentrations tested, with MN frequencies remaining comparable to the control (*p* > 0.05). Similarly, neither free carvacrol (CV) nor the CV-loaded nanoemulsions (NE-CV and NE-CV/VitE) induced a statistically significant increase in micronucleus frequencies at concentrations up to 50 μg/mL.

The cytotoxic potential was assessed using the Cytokinesis-Block Proliferation Index (CBPI) and the percentage of cytostasis across a concentration range of 10, 25, 50, and 100 μg/mL. A critical observation regarding dose-limiting toxicity was made at the highest concentration; specifically, preliminary screening at 100 μg/mL revealed extensive cytotoxicity (CBPI = 1.0; Cytostasis = 100%) for both free and encapsulated carvacrol. Consequently, this concentration served as the cytotoxic threshold, and genotoxicity was subsequently evaluated at the sub-lethal concentrations of 10, 25, and 50 μg/mL. Notably, the concentration of 50 μg/mL induced a cytotoxicity level that approximated the recommended 55 ± 5% reduction in CBPI values according to OECD guidelines (TG 487) [47], thereby validating its selection as the highest scorable dose for the assay.

Initial dose-range finding experiments tested concentrations of 10, 25, 50, and 100 μg/mL to establish the cytotoxicity threshold. Based on these results, genotoxicity was formally evaluated at 10, 25, and 50 μg/mL, as 100 μg/mL induced complete cytostasis (CBPI = 1.0) in all CV-containing formulations. Importantly, no genotoxic effects were observed for any formulation at concentrations up to 50 μg/mL, with micronucleus frequencies remaining statistically comparable to negative controls (*p* > 0.05). This defines a safe, non-genotoxic working range of 0–50 μg/mL for all tested formulations.

The reduced cytotoxicity of nanoencapsulated carvacrol compared to its free form (Figure 5) aligns with the emerging understanding that encapsulation can modulate the bioavailability and cellular interaction of essential oil components [44,45]. While free hydrophobic compounds can rapidly partition into and disrupt cellular membranes, causing acute cytotoxic effects, their gradual release from nanoemulsion droplets allows for cellular adaptation and reduces the peak concentration experienced by cells [45].

**Figure 6:**
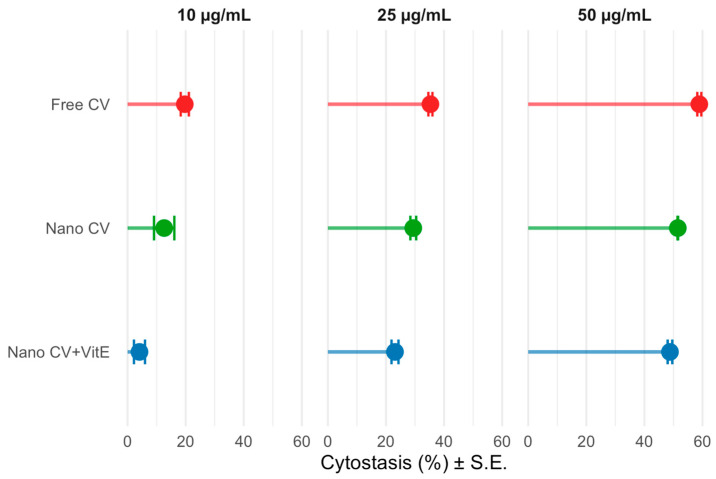
Protective effect of nanoencapsulation against carvacrol-induced cytotoxicity in human lymphocytes. Cytostasis percentages induced by free carvacrol (Free CV), nanoencapsulated carvacrol (Nano CV), and the nanoencapsulated mixture with vitamin E (Nano CV + VitE) at concentrations of 10, 25, and 50 μg/mL (based on active compound content). Values represent mean ± S.E. (n = 3).

Regarding the activity of the samples, a clear dose-dependent increase in cytotoxicity was observed for all carvacrol-containing formulations. Free CV exhibited the most aggressive profile, with cytostasis rising from 19.7% at 10 μg/mL to 58.9% at 50 μg/mL. In contrast, NE-VitE and free VitE showed negligible cytotoxicity across all tested concentrations, confirming the biocompatibility of the antioxidant and the blank carrier matrix. Most importantly, a protective effect was evident upon nanoencapsulation (Figure 5); NE-CV demonstrated reduced cytotoxicity compared to free CV. This reduction in toxicity can be attributed to the controlled release profile of the nanoemulsion. Unlike free carvacrol, which comes into direct, immediate contact with cellular membranes, causing rapid destabilization and lysis, the encapsulated compound is released gradually [45]. This slow-release mechanism may allow cells to metabolize or adapt to the bioactive stressor more effectively, preventing acute cytotoxic shock [44]. Furthermore, the NE-CV/VitE formulation demonstrated the lowest cytotoxicity among the CV-loaded samples (48.8% cytostasis at 50 μg/mL). This enhanced protection likely stems from the synergistic action of VitE. As a potent lipophilic antioxidant, VitE may counteract the oxidative stress often triggered by high concentrations of essential oils, thereby preserving membrane integrity and cell viability [48,49] (Figure 6).

The reduction in cytotoxicity achieved through nanoencapsulation has important practical implications. Free CV exhibits concentration-dependent cytotoxicity, with the CBPI dropping to 1.51 at 50 μg/mL (58.9% cytostasis), approaching the OECD-recommended limit for cytotoxicity assessment. In contrast, NE-CV/VitE maintained a CBPI of 1.63 at the same concentration (48.8% cytostasis), effectively widening the usable concentration window for antimicrobial applications. This expanded therapeutic window means that higher effective doses of CV can be delivered in food coatings without exceeding cytotoxicity thresholds, potentially allowing for enhanced antimicrobial efficacy while maintaining an acceptable safety profile.

### 2.5. Preservation Test in Fresh Minced Pork

To evaluate the practical efficacy of the developed nanoemulsion systems as active food coatings, fresh minced pork was selected as a model perishable food matrix. This section presents a comprehensive assessment of the preservative effects of the NE-CV/VitE coating compared to uncoated controls over a 6-day storage period at 4 °C. The evaluation encompasses multiple quality indicators, including pH evolution (as an indirect measure of microbial spoilage), color stability (a critical sensory attribute), lipid oxidation (TBARS values), heme iron retention, and total viable counts (TVC). Together, these parameters provide a holistic understanding of how the dual-functional coating simultaneously addresses the two primary mechanisms of meat quality deterioration: microbial proliferation and oxidative rancidity.

For the meat preservation study, we selected the co-encapsulated NE-CV/VitE formulation based on its superior multifunctional performance demonstrated in the preceding characterization studies. This formulation exhibited:

1. Optimal physicochemical properties (intermediate particle size of 34.4 nm and enhanced ζ potential of −19.8 mV compared to NE-VitE alone);
2. Preserved antioxidant activity comparable to NE-VitE (EC_50_ 10.76 μL/mL, not statistically different from NE-VitE);
3. Remarkable synergistic antibacterial activity against *E. coli* (requiring only 0.07 mg/mL CV compared to 0.15 mg/mL for NE-CV alone); and
4. An improved safety profile with reduced cytotoxicity compared to free CV.

Furthermore, the antimicrobial efficacy of NE-CV alone as an edible coating for fresh pork has been extensively characterized in our previous publications—including application to minced pork [18] and pork tenderloin [17]—establishing a robust foundation for comparison. The present study therefore focuses on the novel NE-CV/VitE combination to evaluate whether the synergistic benefits observed in vitro translate to enhanced preservation in a real food matrix.

While the meat preservation study initially included five treatment groups (Uncoated, NE-CV, NE-VitE, NE-CV/VitE, and free CV), the detailed time-course data for NE-CV and NE-VitE are presented in Supplementary Materials (Table S8) to maintain focus on the novel co-encapsulated system. A summary of the comparative performance is provided here: NE-CV alone showed antimicrobial efficacy consistent with our previous reports [17,18], achieving a 1.5-log reduction in TVC by day 6, while NE-VitE alone showed minimal antimicrobial effect but reduced TBARS values by approximately 15%. The co-encapsulated NE-CV/VitE outperformed both single-component systems across all quality parameters, confirming the benefit of the combined formulation.

#### 2.5.1. pH and Microbial Growth Dynamics

The evolution of pH in fresh meat during refrigerated storage serves as a reliable indirect indicator of microbial spoilage, as proteolytic bacteria metabolize proteins into alkaline compounds such as ammonia and biogenic amines. Table 4 presents the pH values of uncoated and NE-CV/VitE-coated minced pork samples over the 6-day storage period.

The pH analysis revealed distinct patterns between coated and uncoated samples throughout the 6-day storage period. The uncoated samples exhibited a rapid and significant pH increase from 5.67 ± 0.01 on day 0 to 6.21 ± 0.01 on day 2 (*p* < 0.05), reaching 6.41 ± 0.01 by day 6. This progressive alkalinization is characteristic of meat spoilage and can be attributed to the accumulation of basic nitrogenous compounds, such as ammonia and biogenic amines, produced by microbial proteolytic activity [17,18]. The strong positive correlation between pH increase and TVC values (Table 7) in uncoated samples (R^2^ = 0.98) confirms that microbial proliferation (reaching 8.14 ± 0.01 log CFU/g by day 6) was the primary driver of pH elevation.

In marked contrast, NE-CV/VitE-coated samples maintained remarkably stable pH values throughout storage, with only a minimal increase from 5.65 ± 0.01 to 5.75 ± 0.01 over 6 days (*p* < 0.05). This pH stability directly reflects the effective antimicrobial activity of the coating, as evidenced by the significantly lower TVC values in coated samples (6.12 ± 0.05 log CFU/g at day 6) compared to uncoated controls (8.14 ± 0.01 log CFU/g at day 6). The preservation of near-initial pH values indicates that the NE-CV/VitE coating successfully suppressed the growth of proteolytic spoilage microorganisms responsible for alkaline metabolite production. This finding aligns with the potent antibacterial activity demonstrated against both *E. coli* and *S. aureus* (Table 2), confirming that the antimicrobial efficacy of carvacrol was fully retained within the nanoemulsion system and effectively translated to the complex food matrix. The controlled release properties of the nanoemulsion likely contributed to sustained antimicrobial protection throughout the storage period, preventing the exponential bacterial growth observed in uncoated samples.

#### 2.5.2. Color Stability and Lipid Oxidation

Color is a primary quality attribute that profoundly influences consumer acceptance and purchasing decisions for fresh meat products. To comprehensively evaluate the protective effects of the NE-CV/VitE coating on meat appearance, CIE L*a*b* color parameters were monitored throughout storage, with results presented in Table 5.

The colorimetry results revealed profound differences between coated and uncoated samples, demonstrating the remarkable protective effect of the NE-CV/VitE coating against meat discoloration. Uncoated meat underwent progressive darkening and discoloration, with L* values (lightness) decreasing significantly from 46.49 ± 0.10 at day 0 to 36.55 ± 0.10 at day 6 (*p* < 0.05). This darkening, combined with the significant decline in a* values (redness) from 7.85 ± 0.10 to 4.99 ± 0.30 (*p* < 0.05), indicates extensive myoglobin oxidation and metmyoglobin formation, which imparts the undesirable brown discoloration associated with meat spoilage [20]. The ΔE values, which quantify overall color change relative to fresh meat, decreased dramatically in uncoated samples by day 6 (2.87 ± 0.10), reflecting the complete loss of the characteristic fresh meat appearance.

The NE-CV/VitE-coated samples exhibited remarkably superior color retention throughout storage. L* values initially increased to 55.18 ± 0.10 at day 2, likely due to surface moisture from the coating application, then stabilized at 50.07 ± 0.10 by day 6—significantly higher than uncoated samples (*p* < 0.05). Most notably, a* values (redness) in coated samples showed a transient increase at day 2 (11.40 ± 0.20), possibly due to enhanced oxygen penetration through the hydrophilic coating, promoting initial oxymyoglobin formation, followed by a gradual decline to 6.24 ± 0.20 at day 6, which remained significantly higher than uncoated samples (*p* < 0.05). This superior red color retention directly correlates with the TBARS results (Table 6), establishing a mechanistic link between antioxidant protection and color preservation.

To further elucidate the mechanisms underlying color protection and oxidative stability, lipid oxidation and heme iron content were quantitatively assessed, with results presented in Table 6.

The TBARS analysis revealed that lipid oxidation progressed steadily in all samples during refrigerated storage, but the NE-CV/VitE coating significantly retarded this process. By day 6, coated samples exhibited TBARS values of 0.74 ± 0.01 mg MDA/kg, significantly lower than the 0.82 ± 0.02 mg MDA/kg observed in uncoated controls (*p* < 0.05). Notably, both sample types remained below the sensory threshold of 1.0 mg MDA/kg typically associated with rancidity perception [17], indicating that even uncoated meat remained within acceptable limits for this parameter. However, the statistically significant reduction in TBARS values for coated samples demonstrates the effective antioxidant activity of the incorporated VitE, which functions as a chain-breaking antioxidant by donating hydrogen atoms from its phenolic hydroxyl group to neutralize lipid peroxyl radicals, thereby interrupting the propagation phase of lipid autoxidation [25,29].

The protection of heme iron (Table 6) provides crucial mechanistic insight into the color stabilization observed in Table 5. Heme iron, the central component of myoglobin responsible for the characteristic red color of fresh meat, is highly susceptible to oxidative degradation. Uncoated samples showed a dramatic 40% reduction in heme iron content (from 7.75 ± 0.12 to 4.66 ± 0.33 μg/g) over 6 days, reflecting extensive oxidative damage to the porphyrin ring structure. In contrast, NE-CV/VitE-coated samples retained 71% of their initial heme iron (5.51 ± 0.27 μg/g at day 6), directly attributable to the potent antioxidant activity of VitE (EC_50_ = 8.40 μL/mL in the DPPH assay, Table 1). The strong correlation between heme iron retention, a* value maintenance (R^2^ = 0.94), and TBARS suppression confirms that the antioxidant protection afforded by VitE was fully functional within the nanoemulsion system and effectively preserved both color pigments and lipid fractions from oxidative deterioration. This integrated protection demonstrates that VitE, despite its encapsulation, retained its biological activity and was able to access the lipid phases of the meat matrix, where oxidation primarily occurs.

#### 2.5.3. Integrated Antimicrobial and Antioxidant Performance

The ultimate measure of any food preservation system is its ability to control microbial growth, as microbiological spoilage represents the most immediate and significant threat to meat quality and safety. Table 7 presents the total viable counts (TVC) for uncoated and NE-CV/VitE-coated samples throughout the storage period.

The comprehensive preservation efficacy of the NE-CV/VitE coating can be understood through the complementary and synergistic actions of its two bioactive components. The antibacterial results against foodborne pathogens (Table 2) demonstrate that carvacrol retained its potent antimicrobial activity within the nanoemulsion, with MIC values of 0.15 mg/mL against *E. coli* and 0.30 mg/mL against *S. aureus*. Importantly, the NE-CV/VitE formulation showed enhanced efficacy against *E. coli*, requiring only half the carvacrol concentration (0.07 mg/mL) compared to NE-CV alone (0.15 mg/mL), suggesting a synergistic interaction where VitE may facilitate carvacrol’s membrane-disrupting action by integrating into and perturbing the lipid bilayer of Gram-negative bacteria [32,34].

The translation of this antimicrobial activity to the food matrix is evident in the TVC results (Table 7). Throughout storage, NE-CV/VitE-coated samples maintained significantly lower microbial loads than uncoated controls, with differences exceeding 2 log CFU/g by day 6. This 99% reduction in microbial populations directly explains the preservation of pH stability and the absence of proteolytic spoilage metabolites. The gradual, controlled release of carvacrol from the nanoemulsion matrix likely contributed to sustained antimicrobial protection throughout the 6-day storage period.

The microbiological quality of meat can be assessed against established spoilage thresholds. According to ICMSF (International Commission on Microbiological Specifications for Foods) guidelines, fresh meat is considered acceptable up to 7 log CFU/g, with spoilage becoming evident at 7–8 log CFU/g and organoleptic rejection typically occurring above 8 log CFU/g. Uncoated samples exceeded the 7 log CFU/g spoilage threshold by day 4 (6.79 log CFU/g) and reached organoleptic rejection levels (8.14 log CFU/g) by day 6. In contrast, NE-CV/VitE-coated samples remained below the spoilage threshold throughout the entire 6-day storage period (maximum 6.12 log CFU/g at day 6), representing a substantial extension of microbiological shelf life.

Parallel to antimicrobial protection, the antioxidant activity of VitE (EC_50_ = 8.40 μL/mL in the DPPH assay, Table 1) provided comprehensive oxidative protection. The TBARS results (Table 6) demonstrate that lipid oxidation in coated samples (0.74 ± 0.01 mg MDA/kg at day 6) remained significantly below the threshold of 1.0 mg MDA/kg typically associated with rancidity perception [17]. This antioxidant protection was particularly critical for preserving heme iron and, consequently, meat color. The integrated action of both components is evident in the superior overall quality of coated samples, where antimicrobial protection prevented microbial spoilage while antioxidant protection preserved oxidative stability.

Importantly, the cytotoxicity results (Table 3) confirm that this enhanced preservation efficacy was achieved without compromising safety. NE-CV/VitE demonstrated significantly reduced cytotoxicity compared to free carvacrol (48.8% cytostasis at 50 μg/mL vs. 58.9% for free CV), with VitE likely contributing to this protective effect through its membrane-stabilizing antioxidant properties [48,49]. The absence of genotoxic effects (MN frequencies comparable to the negative control, *p* > 0.05) further confirms the safety profile of the nanoemulsion system.

In conclusion, the NE-CV/VitE coating represents an integrated, multifunctional preservation technology where antimicrobial (carvacrol) and antioxidant (VitE) components work in concert to comprehensively protect meat quality. The nanoemulsion system successfully delivers both bioactivities to the food matrix, providing simultaneous protection against microbial spoilage and oxidative deterioration while maintaining safety and potentially adding nutritional value through VitE enrichment.

## 3. Conclusions

This study successfully developed and characterized novel CSN/LCN-based edible nanoemulsions co-encapsulating carvacrol (CV) and vitamin E (VitE) as multifunctional active coatings for food preservation. The key findings and their implications are summarized below:

Successful Formulation and Physicochemical Stability: The high-speed homogenization technique yielded stable, monodisperse nanoemulsions with droplet sizes in the desirable sub-60 nm range (22.7–57.7 nm). ATR-FTIR analysis confirmed the successful encapsulation of both bioactive compounds without chemical degradation, while revealing intermolecular hydrogen-bonding interactions between components that contribute to system stability. Notably, the combination of CV with VitE in NE-CV/VitE produced an intermediate particle size (34.4 nm) and enhanced zeta potential (−19.8 mV) compared to NE-VitE alone (−9.33 mV), demonstrating that CV positively modulates the interfacial architecture to improve electrostatic stabilization—addressing a common formulation challenge associated with viscous lipophilic antioxidants.

Preserved and Synergistic Bioactivities: The antioxidant evaluation confirmed that VitE retained its potent radical scavenging capacity within the nanoemulsion system (EC_50_ 8.40 µL/mL for NE-VitE), while the co-encapsulated NE-CV/VitE exhibited statistically comparable activity (EC_50_ 10.76 µL/mL), indicating that CV incorporation does not compromise antioxidant performance. More importantly, the antibacterial assessment revealed a remarkable synergistic effect against Gram-negative *E. coli*: NE-CV/VitE achieved complete inhibition at half the carvacrol concentration (0.07 mg/mL) required for NE-CV alone (0.15 mg/mL), suggesting that VitE facilitates CV’s membrane-disrupting action through bilayer perturbation. Against *S. aureus*, antimicrobial activity remained strictly CV dose-dependent, with both formulations requiring 0.30 mg/mL for inhibition, confirming that the Gram-positive cell wall architecture presents a different mechanistic challenge.

Improved Safety Profile Through Nanoencapsulation: The comprehensive toxicological evaluation using the CBMN assay in human lymphocytes provided critical safety validation. While free CV induced dose-dependent cytotoxicity (58.9% cytostasis at 50 μg/mL), nanoencapsulation significantly reduced this effect (48.8% cytostasis for NE-CV/VitE at 50 μg/mL), with VitE likely contributing additional protection through its membrane-stabilizing antioxidant properties. Critically, no genotoxic effects were observed for any formulation at concentrations up to 50 μg/mL, with micronucleus frequencies remaining statistically comparable to negative controls. This safety profile, combined with the absence of antimicrobial activity in blank NEs, confirms that the carrier matrix itself is biocompatible and that all bioactivity derives specifically from the encapsulated compounds.

Comprehensive Preservation Efficacy in a Real Food Matrix: The translational validation on fresh minced pork demonstrated that the NE-CV/VitE coating simultaneously addresses the two primary mechanisms of meat quality deterioration: microbial spoilage and oxidative rancidity. The coating maintained remarkable pH stability (5.65–5.75 over 6 days versus 5.67–6.41 in uncoated samples), directly reflecting effective suppression of proteolytic spoilage microorganisms. This was corroborated by the 2-log reduction in total viable counts at day 6 (6.12 vs. 8.14 log CFU/g for uncoated), representing a 99% reduction in microbial populations. Concurrently, the VitE component provided robust antioxidant protection, significantly reducing lipid oxidation (TBARS 0.74 vs. 0.82 mg MDA/kg at day 6) and, most notably, preserving heme iron content (71% retention vs. 60% in uncoated samples). This heme iron protection directly translated to superior color retention, with coated samples maintaining significantly higher redness (a* values) throughout storage—a critical sensory attribute for consumer acceptance.

Integrated Multifunctional Technology: The NE-CV/VitE system represents a significant advancement beyond conventional single-component active coatings. The formulation achieves three complementary functions: (1) antimicrobial protection via CV-mediated membrane disruption, synergistically enhanced against Gram-negative bacteria by VitE; (2) antioxidant protection via VitE’s chain-breaking radical scavenging; and (3) potential nutritional enrichment through VitE delivery. This multifunctionality addresses the industrial need for comprehensive preservation solutions that can replace multiple synthetic additives with a single, natural, edible system.

Limitations and Future Perspectives: While this study provides comprehensive in vitro and ex vivo validation, several aspects warrant further investigation. The long-term storage stability of the nanoemulsions themselves (>6 months) requires assessment, as does the potential migration behavior of components into different food matrices. Scale-up feasibility and economic analysis for industrial adoption remain to be addressed. Future work should explore the application of this coating system to other perishable food categories (poultry, fish, fresh produce) and investigate potential synergies with additional natural bioactives. The observed differential enhancement against Gram-negative versus Gram-positive bacteria also merits deeper mechanistic investigation using membrane model systems and molecular dynamics simulations. For future investigations, complementary microscopic imaging techniques such as optical microscopy and confocal laser scanning microscopy (CLSM) would provide additional insights into droplet morphology and distribution, particularly for assessing long-term stability and behavior in food matrices

In summary, this study demonstrates that:

- Co-encapsulation of CV and VitE in CSN/LCN nanoemulsions yields stable, sub-60 nm droplets with enhanced physicochemical properties compared to VitE alone
- The combination shows enhanced (additive) antibacterial activity against *E. coli* (50% reduction in CV required) while preserving VitE’s potent antioxidant capacity
- Nanoencapsulation significantly reduces CV cytotoxicity (from 58.9% to 48.8% cytostasis at 50 μg/mL) while maintaining full bioactivity
- NE-CV/VitE coating extends minced pork shelf-life through integrated antimicrobial protection (2-log reduction in TVC) and antioxidant preservation (71% heme iron retention, superior color stability)

In conclusion, this study introduces, for the first time, a CSN/LCN-based nanoemulsion co-encapsulating carvacrol and VitE as an integrated, safe, and highly effective edible coating for food preservation. The system successfully harnesses the complementary bioactivities of its components, demonstrating that strategic combination within a rationally designed nano-delivery system can yield multifunctional performance exceeding that of individual constituents. This work provides a foundation for the development of next-generation natural preservation technologies aligned with bioeconomy principles, consumer preferences for clean-label products, and the growing demand for functional foods with added nutritional value. The demonstrated gel-forming potential of this casein–lecithin nanoemulsion further underscores its suitability as an edible coating, combining the benefits of a fluid precursor with the functionality of a cohesive gel film upon application, in full accordance with the journal’s focus on advanced gel systems.

## 4. Materials and Methods

### 4.1. Materials

Carvacrol (CV, CAS No. 499-75-2), DL-α-Tocopherol (Vitamin E, VitE, CAS No. 59-02-9), sodium caseinate (CAS No. 9005-46-3), and L-α-lectithin from soybean (CAS No. 8002-43-5) were purchased from Sigma-Aldrich Co. (St. Louis, MO, USA). All microbiological media, including Mueller–Hinton agar and broth, and Tryptic Soy Agar (TSA), were also obtained from Sigma-Aldrich. Bacterial cultures of *Escherichia coli* O157:H7 and *Listeria monocytogenes* were acquired from the Institute of Technology of Agricultural Products, ELGO-DEMETER (Lykovryssi, Greece). Fresh pork tenderloin was sourced from a local meat processor.

### 4.2. Preparation of Nanoemulsion (NE) Coatings

Three distinct NE formulations were prepared (Table 8). For each formulation, specified quantities of distilled water, LCN, and sodium caseinate were combined with the respective oil phase (CV, VitE, or their mixture). The mixtures were homogenized using a high-speed ultrasonic homogenizer (15,000 rpm) (Ningbo Scientz Biotechnology Co., Ltd., Ningbo, China) at ambient temperature for 20 min to form stable oil-in-water nanoemulsions.

The selection of a 2% total bioactive concentration (and specifically the 1% CV + 1% VitE combination in NE-CV/VitE) was based on systematic optimization studies from our previous works. In our earlier investigation comparing CV NE versus microemulsions [18], we developed and characterized casein–lecithin-based NEs with 1%, 2%, and 3% carvacrol (data provided in the Supplementary Materials of that study). The 2% formulation exhibited optimal physicochemical properties, including droplet size distribution, physical stability, and encapsulation efficiency, compared to 1% (which showed lower bioactivity) and 3% (which exhibited increased droplet size and reduced stability). Subsequently, in our comparative study of four EO components [17], the 2% CV NE demonstrated high performance among the tested formulations. The present study therefore builds upon these optimized parameters, maintaining the 2% total bioactive load while exploring the synergistic combination of CV and vitE at a 1:1 ratio.

### 4.3. Physicochemical Characterization of NEs

ATR-FTIR Spectroscopy: Chemical characterization and investigation of potential interactions between the nanoemulsion components (CSN, LCN, CV, Vit E) were performed using Attenuated Total Reflectance Fourier-Transform Infrared (ATR-FTIR) spectroscopy. Spectra of pure components and the final NE films (obtained by drying droplets) were recorded on a Shimadzu FT-IRSpirit spectrometer (Shimadzu, Kyoto, Japan) equipped with a diamond ATR crystal. Scans were performed in the range of 4000–400 cm^−1^ at a resolution of 4 cm^−1^. The resulting spectra were analyzed for characteristic functional group peaks and shifts that could indicate encapsulation or molecular interactions within the nanoformulation.

Dynamic Light Scattering (DLS): The hydrodynamic diameter (ζ-average) and polydispersity index (PDI) of the nanoemulsions were measured using a Zetasizer Nano ZS (Malvern Instruments, Malvern, UK). Samples were analyzed at 25 °C. This dilution factor is standard practice for DLS measurements of concentrated emulsions and has been shown to maintain colloidal structure [37]. The results reflect the average droplet size distribution and the homogeneity of the formulations. All measurements were performed in triplicate on three independently prepared samples, with results expressed as mean ± standard deviation.

#### Transmission Electron Microscopy (TEM)

The transmission electron microscope (TEM) study of samples deposited (drop-cast method) on carbon-coated copper grids (CF300-CU-UL, carbon square mesh, CU, 300 mesh from Electron Microscopy Science, Hatfield, PA, USA) was performed using the instrument JEM HR-2100, JEOL Ltd., Tokyo, Japan operated at 200 kV in bright-field mode.

Zeta Potential Measurements: The electrochemical stability of the nanoemulsions was assessed by measuring their zeta potential using the same Zetasizer Nano ZS instrument (Malvern Panalytical Ltd, Malvern, Worcestershire, UK) equipped with a solvent-resistant electrode. Diluted samples (10-fold) were placed in a folded capillary cell, and the zeta potential was determined based on electrophoretic mobility. Highly negative or positive values (typically >|30| mV) indicate stable systems due to strong electrostatic repulsion between droplets.

### 4.4. Determination of Antioxidant Activity

DPPH Radical Scavenging Assay: The antioxidant capacity was evaluated using the 2,2-diphenyl-1-picrylhydrazyl (DPPH) method. Briefly, different volumes of NEs or pure compounds were mixed with a DPPH ethanol solution. After incubation in the dark, the absorbance was measured at 517 nm. The effective concentration required to scavenge 50% of DPPH radicals (EC_50_) was calculated.

ABTS Radical Scavenging Assay: The 2,2′-azino-bis(3-ethylbenzothiazoline-6-sulfonic acid) (ABTS) assay was also performed. An ABTS radical cation solution was mixed with sample aliquots, and the decrease in absorbance at 734 nm was measured after incubation. The EC_50_ value was determined from the dose–response curve.

All assays were performed in quintuplicate (n = 5) for each formulation, with EC_50_ values calculated from dose–response curves.

### 4.5. Evaluation of Antibacterial Activity

#### 4.5.1. Microbial Strains and Culture Conditions

The antimicrobial activity of the developed nanoemulsions was evaluated against two food-borne pathogenic bacteria: the Gram-negative *Escherichia coli* (DSM 1103) and the Gram-positive *Staphylococcus aureus* (DSM 113533). Bacterial strains were routinely cultured on Mueller-Hinton Agar (MHA) (Thermo Scientific™, Waltham, MA, USA) at 37 °C. For the assays, liquid cultures were prepared in Mueller-Hinton Broth (MHB) (Thermo Scientific™, Waltham, MA, USA) and incubated overnight under aerobic conditions.

#### 4.5.2. Determination of Minimum Inhibitory Concentration (MIC)

The MIC was determined by the broth microdilution method in 96-well flat-bottom microplates using resazurin as a cell viability indicator, following the protocol previously described [17,18]. Briefly, serial two-fold dilutions of each nanoemulsion (Blank-NE, NE-VitE, NE-CV, NE-CV/VitE) were prepared in Mueller–Hinton broth, covering a final concentration range from 50% down to approximately 0.10% (*v*/*v*). An inoculum of the test bacterium (*Escherichia coli* or *Staphylococcus aureus*) was added to each well to achieve a final density of approximately 5 × 10^5^ CFU/mL. Plates were incubated at 37 °C for 18 h, after which 50 μL of 0.015% resazurin solution was added, and the plates were re-incubated for 2–3 h. The MIC was defined as the lowest concentration of nanoemulsion that prevented the color change from purple (no growth) to pink (metabolic activity/growth). All assays were performed in triplicate (n = 3) for each bacterial strain and formulation.

#### 4.5.3. Determination of Minimum Bactericidal Concentration (MBC)

The MBC was determined by subculturing aliquots from wells that showed no visible growth (no color change) in the MIC assay, according to the method described in our previous studies [17,18]. A 10 μL volume from each clear well was spot-plated onto Mueller-Hinton agar plates and incubated at 37 °C for 24 h. The MBC was defined as the lowest concentration of nanoemulsion that resulted in no visible colony formation on the agar plates, corresponding to a ≥99.9% reduction in the initial bacterial inoculum. Determinations were performed in triplicate (n = 3) for each bacterial strain and formulation.

MIC and MBC determinations were performed in triplicate (n = 3) for each bacterial strain and formulation

### 4.6. Cytotoxic and Genotoxic Effects of Nanoemulsions in Human Lymphocytes

#### 4.6.1. Ethics Statement and Approval

The experimental use of human lymphocytes adhered to internationally accepted bioethical standards and was approved by the Research Ethics Committee of the University of Patras (Ref. No 11584/6 March 2018).

#### 4.6.2. Whole Blood Collection and Cell Culture Preparation

Whole blood was drawn using heparinized collection tubes from three healthy, non-smoking male donors (aged 20 and 25 years). Donors confirmed no recent history of radiation exposure, pharmacological treatment, or viral infection.

#### 4.6.3. Cytokinesis-Block Micronucleus (CBMN) Assay

The potential cytotoxic and genotoxic effects of the nanoemulsions were evaluated using the CBMN assay in human lymphocytes following established protocols [50,51]. Whole blood from three healthy, non-smoking donors was cultured in Ham’s F-10 medium supplemented with fetal bovine serum and phytohemagglutinin. After 24 h, the nanoemulsions were added to achieve final concentrations of 10, 25, 50, and 100 μg/mL (based on active compound content). Mitomycin C (0.5 μg/mL) served as a positive genotoxic control. At 44 h, cytochalasin-B (6 μg/mL) was added to block cytokinesis, and cultures were harvested after 72 h. Cells were subjected to mild hypotonic treatment, fixed, and stained with 10% Giemsa.

A minimum of 2000 binucleated cells (1000 per donor) were scored per condition. Micronucleus (MN) frequency was expressed in parts per thousand (‰). Cytotoxicity was assessed by calculating the Cytokinesis-Block Proliferation Index (CBPI) and percentage of cytostasis from at least 1000 cells per condition, using formulas described previously [52,53]. All experiments were conducted in triplicate using lymphocytes from three independent donors.

Experiments were conducted in triplicate using lymphocytes from three independent donors, with a minimum of 1000 cells scored per condition.

### 4.7. Application as Active Coatings on Fresh Minced Pork

Fresh minced pork slices (~30 g each) were assigned to five treatment groups: Uncoated (control), NE-CV, NE-VitE, NE-CV/VitE, and a control with free Carvacrol (F-CV). Samples were dip-coated in 25 mL of the respective treatment solution for 2 min, drained, vacuum-packaged, and stored at 4 °C for 9 days. Analyses were performed on days 0, 2, 4, and 6.

### 4.8. Physicochemical and Microbiological Analysis of Coated Meat

#### 4.8.1. pH Analysis

The pH of minced pork samples was measured using a portable pH meter equipped with a penetration electrode and temperature sensor (pH-Star, Matthaus GmbH, Poettmes, Germany), following the procedure described in our previous studies [17,18]. The meter was calibrated with buffer solutions of pH 4.0 and 7.0 at the sample temperature (4 °C). For each treatment group and storage time point (days 0, 2, 4, and 6), ten readings were taken across three independent sample portions. Results are expressed as mean ± standard deviation.

#### 4.8.2. Color Analysis (L*a*b*)

Color parameters (L*, a*, b*) were measured directly on the meat surface using an LS171 colorimeter (Linshang Company, Shenzhen, China) calibrated with a white standard plate, as previously described [17,18]. For each treatment group and storage day, three independent portions were analyzed, with five readings per portion. The total color difference (ΔE) relative to day 0 was calculated as:

Δ*E* = [(*L*^∗^ − *L*_0_^∗^)^2^ + (*a*^∗^ − *a*_0_^∗^)^2^ + (*b*^∗^ − *b*_0_^∗^)^2^]^1/2^

Results are expressed as mean ± standard deviation (n = 3).

#### 4.8.3. Lipid Oxidation (TBARS Assay)

Lipid oxidation was assessed by the thiobarbituric acid reactive substances (TBARS) method according to Shahidi [54,55,56] and our earlier protocols [17,18]. Minced pork (2 g) was mixed with 5 mL of 10% trichloroacetic acid, vortexed, and then 5 mL of 0.02 M aqueous 2-thiobarbituric acid was added. After centrifugation, the absorbance of the supernatant was measured, and TBARS values were calculated using a conversion factor of 3.6. Results are expressed as mg malondialdehyde (MDA) per kg meat (mean ± standard deviation, n = 3).

#### 4.8.4. Heme Iron Content

Heme iron was determined spectrophotometrically following the method of Hornsey [57], as applied in our previous work [17,18]. Briefly, heme pigments were extracted and quantified, and results are expressed as μg heme iron per gram of meat. All measurements were performed in triplicate for each treatment group at each storage time point (mean ± standard deviation).

#### 4.8.5. Microbiological Analysis (Total Viable Counts)

Total viable mesophilic bacteria were enumerated according to ISO 4833-1 [58] and our established protocols [17,18]. Minced pork (10 g) was homogenized with 90 mL of sterile 0.1% peptone water. Serial ten-fold dilutions were prepared, and 0.1 mL aliquots were spread onto Plate Count Agar (PCA). Plates were incubated at 37 °C for 48 h. Results are expressed as log colony forming units per gram (log CFU/g) and represent the mean ± standard deviation of three independent replicates per treatment group and storage time point.

### 4.9. Statistical Analysis

All experiments were conducted in triplicate unless otherwise specified. Data were analyzed using IBM SPSS Statistics (Version 29.0, IBM Corp., Armonk, NY, USA). Results are expressed as mean ± standard deviation (SD) for physicochemical, antioxidant, antibacterial, and meat preservation analyses, or as mean ± standard error (S.E.) for cytotoxicity and genotoxicity assessments.

For comparisons involving multiple groups (antioxidant activity, antibacterial activity across formulations, physicochemical properties of meat over multiple time points, and comparisons between multiple treatment groups), statistical significance was evaluated using One-way Analysis of Variance (ANOVA). When significant differences were detected (*p* < 0.05), post hoc multiple comparisons were performed using Tukey’s Honestly Significant Difference (HSD) test to identify specific differences between treatment groups.

For comparisons involving only two groups (e.g., comparisons between coated and uncoated samples at a single time point in the meat preservation study), statistical significance was assessed using Student’s *t*-test (independent samples, two-tailed).

For the assessment of cytotoxicity and genotoxicity (CBMN assay), data from three independent experiments were analyzed using One-way ANOVA followed by Tukey’s HSD post hoc test to compare treatment groups against controls and to evaluate dose-dependent effects.

## Figures and Tables

**Table 1:** Antioxidant Activity values * (EC_50_) of NEs.

Code Name	EC_50_, DPPH (µL/mL)	EC_50_, ABTS·(µL/mL)
NE-CV	23.21 ± 2.19 ^b^	19.50 ± 2.05 ^b^
NE-VitE	8.40 ± 2.15 ^a^	7.35 ± 1.85 ^a^
NE-CV/VitE	10.76 ± 1.09 ^ab^	9.25 ± 1.30 ^ab^

* Values are presented as mean ± standard deviation (n = 5). Within each column, mean values with different superscript letters (a, b) are significantly different (*p* < 0.05). Statistical analysis was performed using One-way Analysis of Variance (ANOVA) followed by Tukey’s HSD post hoc test.

**Table 2:** Antimicrobial activity (MIC and MBC values) * of nanoemulsions against *E. coli* and *S. aureus*. Values are expressed in both volume percentage (% *v*/*v*) of the nanoemulsion and concentration (mg/mL) of the active compound.

Bacteria	Sample	MIC (%*v*/*v*)	MBC (%*v*/*v*)	Active Compound MIC (mg/mL)	Active Compound MBC (mg/mL)
*E. coli*	Blank	-	-	-	-
	NE-VitE	6.25	6.25	1.18 *	1.18 *
	NE-CV	0.78	0.78	0.15 ^#^	0.15 ^#^
	NE-CV/VitE	0.78	0.78	0.07 ^#^	0.07 ^#^
*S. aureus*	Blank	-	-	-	-
	NE-VitE	-	-	-	-
	NE-CV	1.56	3.13	0.30 ^#^	0.61 ^#^
	NE-CV/VitE	3.13	3.13	0.30 ^#^	0.30 ^#^

(-) No antimicrobial activity; * Concentration refers to VitE; ^#^ Concentration refers to CV. Values are presented as mean ± standard deviation (n = 3). Within each bacterial species, mean values with different superscript symbols are significantly different (*p* < 0.05) according to One-way ANOVA followed by Tukey’s HSD post hoc test.

**Table 3:** Cytotoxic and genotoxic effects of free and nanoencapsulated CV and VitE on human lymphocytes.

Concentrations (μg/mL)	BN	MN ± S.E. (‰)	CPBI ± S.E	Cytostasis (%)
**Control**	1000	2.0 ± 0.0 ^a^	2.23 ± 0.01 ^a^	0
**MMC (0.1)**	1000	67.0 ± 3.3 ^b^	1.65 ± 0.02 ^f^	46.9 ± 1.7
**Blank**	1000	1.5 ± 0.4 ^a^	2.21 ± 0.02 ^a^	1.2 ± 1.2
**CV**				
10	1000	1.0 ± 0.0 ^a^	1.99 ± 0.01 ^cd^	19.7 ± 1.2
25	1000	2.5 ± 0.4 ^a^	1.80 ± 0.01 ^e^	35.3 ± 0.6
50	1000	2.5 ± 0.4 ^a^	1.51 ± 0.01 ^g^	58.9 ± 0.6
100	1000	ND	1.00 ± 0.00 ^h^	100 ± 0.0
**VitE**				
10	1000	1.0 ± 0.0 ^a^	2.23 ± 0.0 ^a^	0.0 ± 0.0
25	1000	2.0 ± 0.0 ^a^	2.20 ± 0.02 ^a^	2.3 ± 1.7
50	1000	1.0 ± 0.0 ^a^	2.20 ± 0.02 ^a^	2.0 ± 1.7
100	1000	1.0 ± 0.0 ^a^	2.19 ± 0.01 ^ab^	3.0 ± 0.6
**NE-CV**				
10	1000	2.0 ± 0.0 ^a^	2.07 ± 0.06 ^bc^	12.6 ± 2.8
25	1000	1.5 ± 0.4 ^a^	1.87 ± 0.02 ^de^	29.4 ± 0.8
50	1000	2.0 ± 0.0 ^a^	1.60 ± 0.00 ^fg^	51.5 ± 0.1
100	1000	ND	1.00 ± 0.00 ^h^	100 ± 0.0
		NE-VitE		
10	1000	1.0 ± 0.0 ^a^	2.22 ± 0.01 ^a^	0.3 ± 0.6
25	1000	1.0 ± 0.0 ^a^	2.22 ± 0.00 ^a^	0.3 ± 0.6
50	1000	1.5 ± 0.4 ^a^	2.21 ± 0.01 ^a^	1.6 ± 0.6
100	1000	1.5 ± 0.4 ^a^	2.20 ± 0.01 ^a^	2.0 ± 0.6
		NE-CV/VitE		
10	1000	2.0 ± 0.0 ^a^	2.18 ± 0.02 ^ab^	4.1 ± 1.6
25	1000	1.5 ± 0.7 ^a^	1.95 ± 0.01 ^cd^	23.1 ± 1.0
50	1000	2.0 ± 0.0 ^a^	1.63 ± 0.01 ^fg^	48.8 ± 0.6
100	1000	ND	1.00 ± 0.00 ^h^	100 ± 0.0

Blank: Blank nanoemulsions; BN: Binucleated lymphocytes; MN: total number of micronuclei in lymphocytes; S.E.: Standard Error; MMC: mitomycin C (positive control); ND: Not determined due to excessive cytotoxicity; Values represent Mean ± Standard Error (S.E.) of three independent experiments.; Different superscript letters (a, b, c.) within the same column indicate statistically significant differences between treatments (*p* < 0.05) Concentrations: For CV, NE-CV, and NE-CV/VitE, concentrations refer to the content of Carvacrol (μg/mL). For VitE and NE-VitE, concentrations refer to the content of VitE (μg/mL). The superscript letters (a, b, ab, bc, de, fg, h, etc.) in the CPBI ± S.E. column indicate statistically significant differences between groups. Values that share the same letter are not significantly different, whereas values with different letters differ significantly (*p* < 0.05, as determined by one-way ANOVA followed by Tukey’s post-hoc test). This notation applies to all superscripts shown, including d, e, f, g, and h.

**Table 4:** pH * analysis of fresh minced pork over time.

Treatment	Day 0	Day 2	Day 4	Day 6
NE-CV/VitE	5.65 ± 0.01 ^Aa^	5.66 ± 0.01 ^Ba^	5.71 ± 0.01 ^Ca^	5.75 ± 0.01 ^Da^
Uncoated	5.67 ± 0.01 ^Aa^	6.21 ± 0.01 ^Bb^	6.34 ± 0.01 ^Cc^	6.41 ± 0.01 ^Dc^

* Values are presented as mean ± standard deviation (n = 3). Different uppercase superscript letters (A–D) within the same row indicate significant differences between storage days (*p* < 0.05). Different lowercase superscript letters (a–c) within the same column indicate significant differences between treatments on the same storage day (*p* < 0.05). Statistical analysis was performed using One-way Analysis of Variance (ANOVA) followed by Tukey’s HSD post hoc test.

**Table 5:** L*a*b* colorimetry analysis * of fresh minced pork over time.

Color Parameter	Storage (Day)	Uncoated	NE-CV/VitE
L*	0	46.49 ± 0.10 ^Ad^	48.61 ± 0.20 ^Ab^
	2	45.54 ± 0.10 ^Bb^	55.18 ± 0.10 ^Ba^
	4	38.97 ± 0.20 ^Cc^	52.41 ± 0.10 ^Cb^
	6	36.55 ± 0.10 ^De^	50.07 ± 0.10 ^Dc^
a*	0	7.85 ± 0.10 ^Ab^	6.68 ± 0.10 ^Aa^
	2	6.34 ± 0.20 ^Ad^	11.40 ± 0.20 ^Ba^
	4	6.02 ± 0.20 ^Ac^	8.31 ± 0.10 ^Cb^
	6	4.99 ± 0.30 ^Bc^	6.24 ± 0.20 ^Db^
b*	0	3.31 ± 0.20 ^Ab^	4.58 ± 0.20 ^Aa^
	2	7.56 ± 0.10 ^Bc^	9.96 ± 0.20 ^Bb^
	4	8.13 ± 0.40 ^Bb^	6.05 ± 0.30 ^Cc^
	6	9.27 ± 0.30 ^Cc^	10.07 ± 0.10 ^Da^
ΔE	2	4.61 ± 0.10 ^Ab^	8.10 ± 0.10 ^Aa^
	4	6.60 ± 0.10 ^Bb^	5.70 ± 0.10 ^Ba^
	6	2.87 ± 0.10 ^Cc^	6.82 ± 0.10 ^Ca^

* Values are presented as mean ± standard deviation (n = 3). Different uppercase superscript letters (A–D) within the same column for each parameter indicate significant differences between storage days (*p* < 0.05). Different lowercase superscript letters (a–e) within the same row indicate significant differences between treatments on the same storage day (*p* < 0.05). Statistical analysis was performed using One-way Analysis of Variance (ANOVA) followed by Tukey’s HSD post hoc test. L*: lightness (0 = black, 100 = white); a*: redness (positive values = red, negative values = green); b*: yellowness (positive values = yellow, negative values = blue); ΔE: total color difference calculated relative to day 0.

**Table 6:** TBARS and heme iron values * of minced pork during storage.

Parameter	Treatment	Day 0	Day 2	Day 4	Day 6
TBARS (mg MDA/kg)	uncoated	0.40 ± 0.01 ^Aa^	0.59 ± 0.02 ^Ba^	0.73 ± 0.02 ^Ca^	0.82 ± 0.02 ^Da^
	NE-CV/VitE	0.40 ± 0.01 ^Aa^	0.55 ± 0.01 ^Bb^	0.64 ± 0.01 ^Cb^	0.74 ± 0.01 ^Db^
Heme iron (μg/g)	uncoated	7.75 ± 0.12 ^Aa^	6.25 ± 0.36 ^Ba^	5.51 ± 0.18 ^Ca^	4.66 ± 0.33 ^Da^
	NE-CV/VitE	7.75 ± 0.12 ^Aa^	7.43 ± 0.12 ^Ab^	6.63 ± 0.21 ^Bb^	5.51 ± 0.27 ^Cb^

* Values are presented as mean ± standard deviation (n = 3). Different uppercase superscript letters (A–D) within the same row indicate significant differences between storage days (*p* < 0.05). Different lowercase superscript letters (a, b) within the same column for each parameter indicate significant differences between treatments on the same storage day (*p* < 0.05). Statistical analysis was performed using One-way Analysis of Variance (ANOVA) followed by Tukey’s HSD post hoc test. TBARS: thiobarbituric acid reactive substances, expressed as mg malondialdehyde per kg meat.

**Table 7:** Total Viable Counts (TVC) * of minced pork during storage (log CFU/g).

Treatment	Day 0	Day 2	Day 4	Day 6
Uncoated	4.39 ± 0.03 ^Aa^	5.59 ± 0.13 ^Ba^	6.79 ± 0.06 ^Ca^	8.14 ± 0.01 ^Da^
NE-CV/VitE	4.39 ± 0.03 ^Aa^	4.81 ± 0.04 ^Bb^	5.52 ± 0.07 ^Cb^	6.12 ± 0.05 ^Db^

* Values are presented as mean ± standard deviation (n = 3). Different uppercase superscript letters (A–D) within the same row indicate significant differences between storage days (*p* < 0.05). Different lowercase superscript letters (a, b) within the same column indicate significant differences between treatments on the same storage day (*p* < 0.05). Statistical analysis was performed using One-way Analysis of Variance (ANOVA) followed by Tukey’s HSD post hoc test. TVC: Total Viable Counts expressed as log colony forming units per gram.

**Table 8:** Composition of the prepared nanoemulsion (NE) coatings. All values are presented as grams (g) or milliliters (mL) per 100 mL final volume (%, *w*/*v*). The bioactive compounds (CV and VitE) themselves constitute the oil phase; no additional carrier oil was used.

Sample Description	Code Name	H_2_O (mL)	LCN (g)	CSN (g)	CV (mL)	VitE (mL)
Nanoemulsion with 2% CV	NE-CV	94.0	0.5	4	2.0	0
Nanoemulsion with 2% VitE	NE-VitE	94.0	0.5	4	0	2.0
Nanoemulsion with 1% CV + 1% VitE	NE-CV/VitE	94.0	0.5	4	1.0	1.0

Note: The total volume sums to 100 mL (water) plus the mass/volume of emulsifiers and bioactives. The final nanoemulsions are oil-in-water systems where CV and VitE comprise the dispersed oil phase, stabilized by CSN and LCN.

## Data Availability

The datasets generated for this study are available on request to the corresponding author.

## Supplementary Materials

The following supporting information can be downloaded at: https://www.mdpi.com/article/10.3390/gels12040300/s1, Table S1: Detailed ATR-FTIR band assignments for pure components and nanoemulsion formulations; Table S2: Polydispersity Index (PDI) values of prepared nanoemulsion formulations; Table S3: Five Replicate EC_50_, DPPH and EC_50_, ABTS values for each sample and assay; Table S4: Extended pH dataset for minced pork during storage; Table S5: Extended colorimetry (L*a*b*) dataset for minced pork; Table S6: Extended TBARS dataset for minced pork (mg MDA/kg); Table S7: Extended heme iron dataset for minced pork (μg/g); Table S8: Extended total viable counts (TVC) dataset for minced pork (log CFU/g).
