# Supplementary material for: Casein–Lecithin Nanoemulsions Co-Encapsulating Vitamin E and Carvacrol as Multifunctional Edible Coatings for Meat Preservation
Source: Gels. 2026 Apr 1;12(4):300. doi: 10.3390/gels12040300 (PMC13116382; doi:10.3390/gels12040300)
Supplement: Supplementary file 1 [file gels-12-00300-s001.zip › gels-4199133-supplementary.pdf]

Supplementary material file for:

Casein–Lecithin Nanoemulsions Co-Encapsulating Vitamin E and Carvacrol as Multifunctional Edible Coatings for Meat Preservation

Aris E. Giannakas [1,*], Achilleas Kechagias [1], Margarita Dormousoglou [2], Georgia Karakasidou [1], Dimitrios Moschovas [3], Eleni Triantafyllou [3], Areti A. Leontiou [1], Andreas Giannakas [1], Panagiota Stathopoulou [2], Apostolos Avgeropoulos [3] and Constantinos E. Salmas [3,*]

[1] Department of Food Science and Technology, University of Patras, 30100 Agrinio, Greece; up1110842@upatras.gr (A.K.); up1091836@ac.upatras.gr (G.K.); aleontiu@upatras.gr (A.A.L.); andgiannakas@upatras.gr (A.G.)
[2] Department of Sustainable Agriculture, University of Patras, 30100 Agrinio, Greece; m.dormousoglou@upatras.gr (M.D.); panstath@upatras.gr (P.S.)
[3] Department of Materials Science Engineering, University of Ioannina, Dourouti, 45110 Ioannina, Greece; dmoschov@uoi.gr (D.M.); triantafyllou.eleni@uoi.gr (E.T.); aavger@uoi.gr (A.A.)
* Correspondence: agiannakas@upatras.gr (A.E.G.); ksalmas@uoi.gr (C.E.S.)

Table S1. Detailed ATR-FTIR band assignments for pure components and nanoemulsion formulations.

Component	Wavenumber (cm^{-1})	Assignment	Reference
**Vitamin E (VitE)**	~3550-3200	O-H stretching (phenolic hydroxyl)	[25,30]
	~2955-2950	Asymmetric CH_3 stretching	[25]
	~2865-2860	Symmetric CH_3 stretching	[25]
	~1465-1450	CH_2 scissoring + asymmetric CH_3 bending	[25]
	~1380-1370	Symmetric CH_3 bending (umbrella, gem-dimethyl)	[25]
	~1270-1260	C-O stretching (phenolic Ar-OH) + in-plane O-H bending	[25,35]
	~1090-1080	Aromatic in-plane C-H bending	[25,35]
**Carvacrol (CV)**	~3400-3200	O-H stretching (phenolic hydroxyl)	[36,37]
	~3050-3000	Aromatic =C-H stretching	[36,37]
	~2960-2850	Aliphatic C-H stretching	[36,37]

	~1610-1580	Aromatic C=C stretching	[36,37]
	~1510-1490	Aromatic C=C stretching	[36,37]
	~1460-1440	CH_2 bending	[36,37]
	~1380-1360	CH_3 symmetric bending	[36,37]
	~1270-1250	C-O stretching (phenolic OH)	[36,37]
	~830-810	C-H out-of-plane bending (1,2,4-trisubstituted benzene ring)	[36,37]
**Casein (CSN)**	~3280	N-H stretching (amide A)	[38,39]
	~1650	C=O stretching (amide I)	[38]
	~1540	N-H bending + C-N stretching (amide II)	[39]
	~1450	CH_2 scissoring (aliphatic side chains)	[40]
	~1395	COO^- symmetric stretching (carboxylate groups)	[40]
	~1235	C-N stretching + N-H bending (amide III)	[40]
**Lecithin (LCN)**	~3300-3200	O-H and N-H stretching (phosphatidylcholine headgroup + hydration water)	[41,42]
	~2920	Asymmetric CH_2 stretching (fatty acid chains)	[43,44]
	~2850	Symmetric CH_2 stretching (fatty acid chains)	[43,44]
	~1735	C=O stretching (ester carbonyl, glycerol backbone)	[45]
	~1240	P=O asymmetric stretching (phosphate moiety)	[45]
	~1080	P-O-C symmetric stretching (phosphate moiety)	[45]
	~970	$N^+(CH_3)_3$ symmetric stretching (choline headgroup)	[41]
**NE Formulations**	~3300-3200	Broadened/enhanced O-H/N-H stretching (increased H-bonding)	[46,47]
	~1650	Amide I (slight shift in NE-CV and NE-CV/VitE indicating interaction with CV)	[48]
	~1270-1260	Phenolic C-O stretch (CV and VitE) - retained, confirming preservation	This study

	~1240, ~1080	Phosphate bands (LCN) - minor shifts indicating interactions with CSN	[49]

Table S2. Polydispersity Index (PDI) values of prepared nanoemulsion formulations.

Formulation	PDI Value
**NE-CV (2% Carvacrol)**	0.495
**NE-VitE (2% Vitamin E)**	0.489
**NE-CV/VitE (1% CV + 1% VitE)**	0.664

Values represent the polydispersity index determined by Dynamic Light Scattering (DLS) at 25°C. All measurements were performed in triplicate within 48 h of preparation.

Table S3 Five Replicate EC50,DPPH, and EC50,ABTS Values for Each Sample and Assay

Code Name	EC_{50},DPPH·(μl/ml) Replicates	EC_{50},ABTS·(μl/ml) Replicates
**NE-CV**	25.80, 22.05, 23.50, 21.10, 23.60	21.80, 18.95, 19.45, 17.60, 19.70
**NE-VitE**	10.90, 7.80, 8.05, 6.50, 8.75	9.60, 6.95, 7.20, 5.40, 7.60
**NE-CV/VitE**	12.00, 10.20, 10.85, 9.50, 11.25	10.70, 8.80, 9.30, 8.10, 9.40

Table S4. Extended pH Dataset for Minced Pork During Storage

Treatment	Replicate	Day 0	Day 2	Day 4	Day 6
**NE-CV/VitE**	R1	5.64	5.65	5.70	5.74
	R2	5.65	5.66	5.71	5.75
	R3	5.66	5.67	5.72	5.76
**Mean ± SD**		**5.65 ± 0.01**	**5.66 ± 0.01**	**5.71 ± 0.01**	**5.75 ± 0.01**
**Uncoated**	R1	5.66	6.20	6.33	6.40
	R2	5.67	6.21	6.34	6.41
	R3	5.68	6.22	6.35	6.42
**Mean ± SD**		**5.67 ± 0.01**	**6.21 ± 0.01**	**6.34 ± 0.01**	**6.41 ± 0.01**

Table S5. Extended Colorimetry (L*a*b*) Dataset for Minced Pork

Treatment	Parameter	Replicate	Day 0	Day 2	Day 4	Day 6
**NE-CV/VitE**	**L***	R1	48.41	55.08	52.31	49.97
		R2	48.61	55.18	52.41	50.07
		R3	48.81	55.28	52.51	50.17
	**Mean ± SD**		**48.61 ± 0.20**	**55.18 ± 0.10**	**52.41 ± 0.10**	**50.07 ± 0.10**
	**a***	R1	6.58	11.20	8.21	6.04
		R2	6.68	11.40	8.31	6.24
		R3	6.78	11.60	8.41	6.44
	**Mean ± SD**		**6.68 ± 0.10**	**11.40 ± 0.20**	**8.31 ± 0.10**	**6.24 ± 0.20**
	**b***	R1	4.38	9.76	5.75	9.97
		R2	4.58	9.96	6.05	10.07
		R3	4.78	10.16	6.35	10.17
	**Mean ± SD**		**4.58 ± 0.20**	**9.96 ± 0.20**	**6.05 ± 0.30**	**10.07 ± 0.10**
	**ΔE**	R1	-	8.00	5.60	6.72
		R2	-	8.10	5.70	6.82
		R3	-	8.20	5.80	6.92
	**Mean ± SD**		**-**	**8.10 ± 0.10**	**5.70 ± 0.10**	**6.82 ± 0.10**
**Uncoated**	**L***	R1	46.39	45.44	38.77	36.45
		R2	46.49	45.54	38.97	36.55
		R3	46.59	45.64	39.17	36.65
	**Mean ± SD**		**46.49 ± 0.10**	**45.54 ± 0.10**	**38.97 ± 0.20**	**36.55 ± 0.10**
	**a***	R1	7.75	6.14	5.82	4.69
		R2	7.85	6.34	6.02	4.99

	R3	7.95	6.54	6.22	5.29
**Mean ± SD**		**7.85 ± 0.10**	**6.34 ± 0.20**	**6.02 ± 0.20**	**4.99 ± 0.30**
**b***	R1	3.11	7.46	7.73	8.97
	R2	3.31	7.56	8.13	9.27
	R3	3.51	7.66	8.53	9.57
**Mean ± SD**		**3.31 ± 0.20**	**7.56 ± 0.10**	**8.13 ± 0.40**	**9.27 ± 0.30**
**ΔE**	R1	-	4.51	6.50	2.77
	R2	-	4.61	6.60	2.87
	R3	-	4.71	6.70	2.97
**Mean ± SD**		**-**	**4.61 ± 0.10**	**6.60 ± 0.10**	**2.87 ± 0.10**

Table S6. Extended TBARS Dataset for Minced Pork (mg MDA/kg)

Treatment	Replicate	Day 0	Day 2	Day 4	Day 6
**CONTROL**	R1	0.39	0.57	0.71	0.80
	R2	0.40	0.59	0.73	0.82
	R3	0.41	0.61	0.75	0.84
**Mean ± SD**		**0.40 ± 0.01**	**0.59 ± 0.02**	**0.73 ± 0.02**	**0.82 ± 0.02**
**NE-CV/VitE**	R1	0.39	0.54	0.63	0.73
	R2	0.40	0.55	0.64	0.74
	R3	0.41	0.56	0.65	0.75
**Mean ± SD**		**0.40 ± 0.01**	**0.55 ± 0.01**	**0.64 ± 0.01**	**0.74 ± 0.01**

Table S7. Extended Heme Iron Dataset for Minced Pork (μg/g)

Treatment	Replicate	Day 0	Day 2	Day 4	Day 6
**CONTROL**	R1	7.63	5.89	5.33	4.33
	R2	7.75	6.25	5.51	4.66
	R3	7.87	6.61	5.69	4.99
**Mean ± SD**		**7.75 ± 0.12**	**6.25 ± 0.36**	**5.51 ± 0.18**	**4.66 ± 0.33**
**NE-CV/VitE**	R1	7.63	7.31	6.42	5.24
	R2	7.75	7.43	6.63	5.51
	R3	7.87	7.55	6.84	5.78
**Mean ± SD**		**7.75 ± 0.12**	**7.43 ± 0.12**	**6.63 ± 0.21**	**5.51 ± 0.27**

Table S8. Extended Total Viable Counts (TVC) Dataset for Minced Pork (log CFU/g)

Treatment	Replicate	Day 0	Day 2	Day 4	Day 6
**Uncoated**	R1	4.36	5.46	6.73	8.13
	R2	4.39	5.59	6.79	8.14
	R3	4.42	5.72	6.85	8.15
**Mean ± SD**		**4.39 ± 0.03**	**5.59 ± 0.13**	**6.79 ± 0.06**	**8.14 ± 0.01**
**NE-CV/VitE**	R1	4.36	4.77	5.45	6.07
	R2	4.39	4.81	5.52	6.12
	R3	4.42	4.85	5.59	6.17
**Mean ± SD**		**4.39 ± 0.03**	**4.81 ± 0.04**	**5.52 ± 0.07**	**6.12 ± 0.05**
